# Artificial local magnetic field inhomogeneity enhances *T*_2_ relaxivity

**DOI:** 10.1038/ncomms15468

**Published:** 2017-05-18

**Authors:** Zijian Zhou, Rui Tian, Zhenyu Wang, Zhen Yang, Yijing Liu, Gang Liu, Ruifang Wang, Jinhao Gao, Jibin Song, Liming Nie, Xiaoyuan Chen

**Affiliations:** 1 State Key Laboratory of Molecular Vaccinology and Molecular Diagnostics & Center for Molecular Imaging and Translational Medicine, School of Public Health, Xiamen University, Xiamen 361102, China; 2 Laboratory of Molecular Imaging and Nanomedicine, National Institute of Biomedical Imaging and Bioengineering, National Institutes of Health, Bethesda, Maryland 20892, USA; 3 Department of Electronic Science and Fujian Key Laboratory of Plasma and Magnetic Resonance, Xiamen University, Xiamen 361005, China; 4 State Key Laboratory of Physical Chemistry of Solid Surfaces, The Key Laboratory for Chemical Biology of Fujian Province, and Department of Chemical Biology, College of Chemistry and Chemical Engineering, Xiamen University, Xiamen 361005, China

## Abstract

Clustering of magnetic nanoparticles (MNPs) is perhaps the most effective, yet intriguing strategy to enhance *T*_2_ relaxivity in magnetic resonance imaging (MRI). However, the underlying mechanism is still not fully understood and the attempts to generalize the classic outersphere theory from single particles to clusters have been found to be inadequate. Here we show that clustering of MNPs enhances local field inhomogeneity due to reduced field symmetry, which can be further elevated by artificially involving iron oxide NPs with heterogeneous geometries in terms of size and shape. The *r*_2_ values of iron oxide clusters and Landau–Lifshitz–Gilbert simulations confirmed our hypothesis, indicating that solving magnetic field inhomogeneity may become a powerful way to build correlation between magnetization and *T*_2_ relaxivity of MNPs, especially magnetic clusters. This study provides a simple yet distinct mechanism to interpret *T*_2_ relaxivity of MNPs, which is crucial to the design of high-performance MRI contrast agents.

## Abstract (web summary)

The signal detected in magnetic resonance imaging comes from the relaxation of proton nuclear magnetization. Here, Zhou *et al*. introduce magnetic field inhomogeneity as a parameter to design iron oxide nanoparticle clusters to enhance the relaxation rate of nearby protons, thereby increasing image contrast.

Magnetic nanoparticles (MNPs) have received tremendous attention in a variety of fields such as data storage^1^, biochemical analysis^2^,^3^ and biomedicine^4^,^5^. Magnetic resonance imaging (MRI) is a powerful and non-invasive technique to detect lesions in soft tissue^6^; however, it often suffers from limited contrast that has encouraged the exploitation of MNPs as MRI contrast agents^7^,^8^,^9^. To pursue high-performance MRI contrast agents, the outersphere motional averaging regime (MAR) theory has been applied to guide the rational engineering of MNPs^10^,^11^. In short, MAR theory illustrates that *T*_2_ contrast efficiency (*r*_2_) is related to the diffusion of surrounding water protons and the physical properties of MNPs, which can be tuned by their size^12^,^13^, shape^14^,^15^,^16^,^17^,^18^, component^19^,^20^,^21^, crystal structure^22^,^23^ and surface property^24^,^25^.

Another intriguing phenomenon is that cluster formation from single MNPs can cause a marked decrease of *T*_2_ relaxation times and thus increase of *r*_2_ values, or vice versa^26^. On this basis, magnetic resonance switch (MRSw) systems have been developed to detect a wide range of targets, from metal ions to tumour cells^27^,^28^,^29^. The *T*_2_ relaxivity of magnetic clusters is dependent on a number of parameters such as size, frame composition and water penetrating behaviour^30^,^31^. However, the underlying mechanism of the clustering effect is not clear and the attempts to generalize the principles of single-particle relaxivity to clusters have been confronted with a great deal of complications^26^. On clustering, for example, it is assumed that the decreased surface-to-volume ratio may attenuate the effective radius compared with that of isolated single MNPs. Moreover, the long-range order of magnetic spins during the formation of aggregated clusters is considerably preserved at the same level, which deprives the possibility to enhance the magnetization of clusters^32^. Considerable attention has been focused on controlling the self-assembly of MNPs into clusters, while having variable *r*_2_ values with large deviations^33^,^34^,^35^. The exploitation of this prevailing effect, especially the potential mechanism behind it, is thus appealing to guide the rational design of magnetic clusters for MRI and MRSw sensing applications.

Diffusion of protons around the local magnetic field induced by MNPs may lead to the loss of proton spin coherence, namely dephasing, which forms the fundamental basis of *T*_2_ contrast agents^36^,^37^. Although manufacturers have taken great efforts to make a uniform magnet, the magnetic field in an MRI machine is still not perfectly uniform. The considerable level of field inhomogeneity gives rise to *T*_2_* decay from the intrinsic inhomogeneous magnet. This requires a spin-echo array to refocus the signal because *T*_2_* decay is an order-of-magnitude faster than the intrinsic *T*_2_ decay by atomic and molecular mechanisms^6^. Encouraged by this rationale, MNPs have been developed as *T*_2_ contrast agents due to the ability to induce local field inhomogeneity^38^.

It has been partially identified that the saturated magnetization (*M*_s_) value is directly proportional to the *T*_2_ relaxivity (*r*_2_) of MNPs in MAR; however, the prediction of *r*_2_ from the *M*_s_ value is based on the averaged behaviour of proton dephasing around given MNPs^10^,^11^. Although the relationship between proton *T*_2_ dephasing and *M*_s_ value has been well established in single-domain MNPs, it is still not clear how proton dephasing is related to the *M*_s_ value of multi-domain magnetic clusters. In addition, the static dephasing regime (SDR)^39^ and echo-limiting regime^36^ mechanisms take part when the size of MNPs exceeds the threshold of MAR, further complicating the understanding of *T*_2_ relaxivity in magnetic clusters. Here we show that the *T*_2_ relaxivity of MNPs can be linked to the local magnetic field inhomogeneity induced by MNPs. Moreover, we are able to artificially tune the local magnetic field inhomogeneity of clusters through integrating multicomponent single MNPs with different geometries in terms of size and shape, thus increasing the *T*_2_ relaxivity of clusters.

## Results

### Rationale

**Figure 1:**
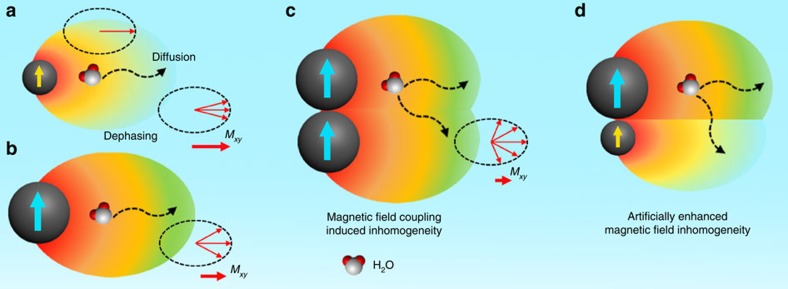
Proton diffusion and dephasing around magnetic nanoparticles. (**a**,**b**) Magnetic nanoparticles (MNPs) with a larger size or magnetization generate a higher degree of magnetic field gradient, leading to higher level of proton dephasing when water molecules diffuse around MNPs. *M*_*xy*_ represents the net nuclei spins on the transverse plane. Dashed arrows indicate the proposed route for water molecule diffusion and dephasing. (**c**) The magnetic field coupling between adjacent MNPs increases the level of field inhomogeneity, which confers greatly enhanced effect to the loss of phase coherence. (**d**) Magnetic field inhomogeneity can be further elevated by artificially involving MNPs of different sizes in proximity, owing to the reduced magnetic field symmetry around MNPs.

Magnetization of MNPs induces local inhomogeneity of magnetic field that is related to the *M*_s_ value of MNPs. To gain broader significance, we reason that the *M*_s_ value can be solved as induced field inhomogeneity, and that adjacent MNPs may generate higher degrees of local field complexity over single MNPs due to the reduced symmetry of local magnetic field. Therefore, it is assumed that the increased level of field inhomogeneity may largely enhance the perturbation of proton phase coherence as water molecules diffuse around adjacent MNPs (Fig. 1), which may be the direct reason for the enhanced *T*_2_ relaxivity of magnetic clusters. In light of the fact that non-spherical MNPs show enhanced *T*_2_ relaxivity compared to spherical ones with equivalent solid volume^14^,^15^, it is natural to assume the role of shape anisotropy in inducing local field inhomogeneity. To further test this assumption, we speculate that field inhomogeneity can be artificially enhanced through assembling different MNPs with heterogeneous local field geometries. We employed iron oxide NPs (IO NPs) with different sizes and shapes as models. The heterogeneity in field geometry may greatly reduce the symmetry and augment the inhomogeneity of the induced local magnetic field, thus conferring a strong influence on the *T*_2_ relaxation shortening effect (Fig. 1).

### MRI performance of iron oxide clusters

**Figure 2:**
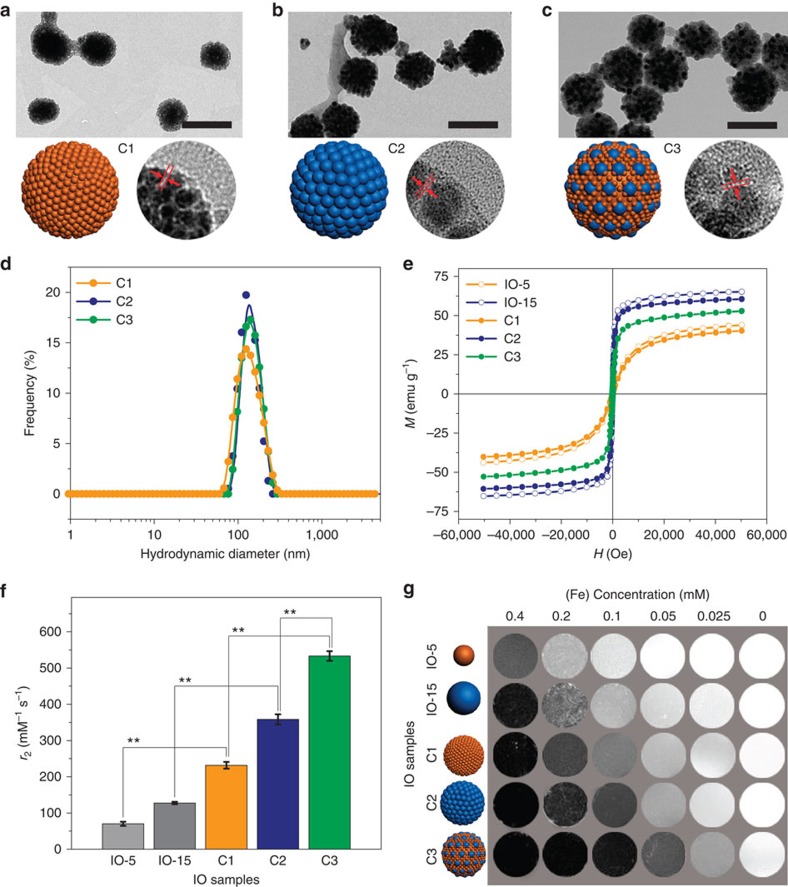
Iron oxide clusters C1–C3. (**a**–**c**) Transmission electron microscopy (TEM) and high-resolution TEM images, as well as cartoons of iron oxide (IO) clusters C1–C3, respectively. Scale bars, 200 nm. The inter-particle distances (*L*) are around 1 nm. (**d**) Dynamic light scattering (DLS) measurements indicate uniform hydrodynamic diameters of IO clusters C1–C3. (**e**) Magnetic hysteresis (*M*–*H*) curves of single IO-5 and IO-15 NPs, and IO clusters C1–C3 measured at 300 K. (**f**) Columns show *r*_2_ values of IO clusters C1–C3, as well as the single IO-5 and IO-15 NPs. Mean values±s.d.; *n*=3 (**P<0.01). (**g**) MR phantom of different IO samples show concentration- and relaxivity-dependent contrasts. Darker contrast indicates stronger *T*_2_ relaxation enhancement for each concentration. All MRI studies were conducted on a 7 T MRI scanner.

We employed two types of IO NPs with average diameters of 5.2 and 15.1 nm as units to explore the clustering effect on *T*_2_ relaxivity, denoted as single IO-5 and IO-15 NPs (Supplementary Methods), respectively. By finely controlling the synthetic conditions, we show that both IO-5 and IO-15 are monodispersed with small size deviations of 4.4% and 2.9%, respectively (Supplementary Fig. 1). The IO NPs were pre-decorated with poly(methyl methacrylate) (PMMA) and polyethylene glycol (PEG) to allow for self-assembly through a similar process as reported previously^40^. To obtain IO clusters with different components, IO-5 only, IO-15 only, and IO-5 plus IO-15 were used as building blocks to form IO clusters C1, C2 and C3, respectively. The ratio of IO-5 and IO-15 NPs in IO cluster C3 was set as 1:1 with respect to iron mass. Transmission electron microscopy (TEM) and scanning electron microscopy images show uniform spheres with diameters of 115.5±10.4, 127.8±13.4 and 129.2±11.2 nm for C1, C2 and C3, respectively (Fig. 2a–c; Supplementary Fig. 2). The inter-particle distances (*L*) were ∼1.0 nm as revealed by high-resolution TEM images (Fig. 2a–c). Dynamic light scattering analysis revealed similar hydrodynamic diameters of 134.3±37.2, 136.9±26.8 and 151.5±28.1 nm for C1, C2 and C3, respectively (Fig. 2d). It is noteworthy that the IO clusters are stable in aqueous solution for at least 45 days without obvious agglomeration or change in hydrodynamic diameter (Supplementary Fig. 3), probably due to the PEG decorated surface on the IO clusters. Magnetic hysteresis (*M*–*H*) curves measured at 300 K indicate that both single IO-5 and IO-15 NPs exhibit typical superparamagnetism, but with different saturation magnetization (*M*_s_) values of 43.18 and 65.10 e.m.u. g^−1^ of NP weight, respectively (Fig. 2e). Both the size and saturation magnetization are directly proportional to the *T*_2_ relaxivity of the IO-5 and IO-15 NPs. On clustering, IO clusters C1–C3 exhibit slightly lower *M*_s_ values than their single components, which could be attributed to two factors including the weight proportion of non-magnetized organic polymers in clusters and the demagnetization effect by intra-cluster dipolar interactions^41^. On the other hand, the intra-cluster dipolar interactions by the formation of IO clusters would enhance the dynamic magnetic response of clusters over single NPs^41^. By changing the molecular weight of PMMA, we also obtained IO clusters C4 and C5 with similar components as C3, but with different inter-particle distances *L* of about 0.1 and 5.0 nm, respectively (Supplementary Fig. 4).

To evaluate the *T*_2_ MRI performance, we measured the *r*_2_ values of single IO NPs and IO clusters on a 7 T MRI scanner. The *r*_2_ values of IO clusters C1 and C2 are 231.6±9.3 and 358.3±14.2 mM^−1^ s^−1^, respectively (Fig. 2f; Supplementary Table 1). These values are approximately three-fold higher than those of single IO-5 and IO-15 NPs, 70.2±5.7 and 127.4±3.4 mM^−1^ s^−1^, respectively (**P<0.01). Here we found that the *r*_2_ value of C3 is increased to 533.4±13.2 mM^−1^ s^−1^ (**P<0.01), significantly higher than those of C1 and C2 (Fig. 2f), whereas simply mixing monodispersed single IO-5 and IO-15 NPs exhibited averaged *T*_2_ relaxation times compared to each single component at different concentrations (Supplementary Fig. 5). We further prepared IO C3_low_ and C3_high_ samples in which the iron mass ratios are 2:1 and 1:2 (that is, the number ratios are 54:1 and 13.5:1) with respect to IO-5 to IO-15 NPs, respectively (Supplementary Fig. 6). The results showed *r*_2_ values of 295.8±23.9 and 486.6±18.5 mM^−1^ s^−1^ for IO C3_low_ and C3_high_ samples, respectively, indicating a vital role of particle distribution and fraction in producing local field inhomogeneity (that is, *T*_2_ relaxivity). However, the optimal ratio of IO-5 and IO-15 NPs for obtaining the highest *r*_2_ value of IO cluster C3 model needs to be further determined by both experiments and simulations. An MR phantom study confirmed that higher *r*_2_ values lead to more prominent *T*_2_ contrasts even at low iron concentrations (Fig. 2g). On the other hand, the *r*_2_ values of IO clusters C4 and C5 are 515.3±18.9 and 445.8±24.6 mM^−1^ s^−1^, respectively (Supplementary Table 1). Therefore, the inverse correlation between *r*_2_ value and inter-particle distance rules out the influence of water penetration effect on *T*_2_ relaxivity^30^. The decrease of the *r*_2_ value with increased inter-particle distance in C5 could be due to the reduced magnetic field coupling effect between adjacent MNPs attenuate the local field inhomogeneity and/or the enhanced motion of individual particles that is more likely to average out the proton dephasing. These results indicate that the formation of clusters from IO NPs with different sizes and magnetizations could confer intriguing effects on *T*_2_ relaxation enhancement, which is also dependent on the inter-particle distance within clusters.

### Landau–Lifshitz–Gilbert simulations

To investigate the magnetizing features at the nanoscale, we employed Landau–Lifshitz–Gilbert (LLG) algorithms to calculate the stray field and the stray field gradient induced by MNPs^42^,^43^,^44^,^45^. To study the field gradient surrounding the IO NPs, we first calculated the differential of the stray field induced by an IO NP as a function of distance 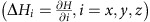. The results showed that only the components of the stray field gradient in the *x* and *z* directions are considered when an external magnetic field is applied along the +*x* direction (Supplementary Fig. 7).

**Figure 3:**
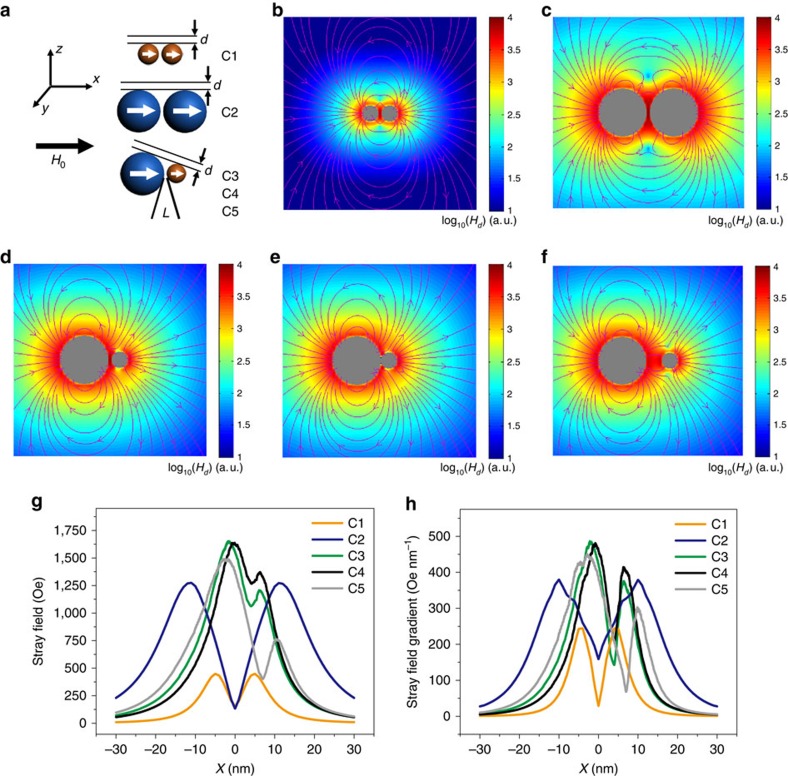
Landau–Lifshitz–Gilbert simulations. (**a**) Cartoon models representing the simulations of two adjacent iron oxide nanoparticles (IO NPs) of different sizes. The inter-particle distance (*L*) is 1.0 nm in models C1–C3. The models C4 and C5 are derived from model C3 but with different *L* of 0.1 and 5.0 nm, respectively. (**b**–**f**) Landau–Lifshitz–Gilbert simulation results of the stray field for model C1–C5, respectively. The *L* is 1 nm (**b**–**d**), 0.1 nm (**e**) and 5.0 nm (**f**). Colour bars represent log_10_(*H*_*d*_) (a.u.), where *H*_*d*_ is the calculated stray field. (**g**,**h**) Calculated stray field and stray field gradient of C1–C5 with an out-of-plane distance (*d*) of 3 nm.

We then showed that single IO-5 and IO-15 NPs exhibit a magnetization-dependent stray field and field gradient around each particle (Supplementary Fig. 8). In light of the outersphere theory, the *M*_s_ value of MNPs is hereby specified as the induced local field inhomogeneity by MNPs, provided that single IO NPs are well dispersed without aggregation. When increasing the particle size, the maximal *r*_2_ value is scaled in the SDR where the diffusion distance of protons is negligible compared with the particle size^39^. The *T*_2_ relaxivity reaches a plateau and is independent of the size of magnetic particles in the SDR. The onset size threshold of the SDR for IO NPs is in the neighborhood of 30–50 nm, depending on a number of parameters such as the particle characteristics (for example, size and magnetization) and experimental parameters of the pulse sequence^15^,^26^. We have obtained IO clusters in the condition of the SDR, but with significantly different *r*_2_ values, which motivated us to revisit the diffusion and dephasing of protons nearby. To simplify, we simulated the stray field and stray field gradient of models with two IO NPs in line with that of IO clusters C1–C5 (Fig. 3a–f and Supplementary Fig. 9). Since proton dephasing at the inner zone of MNPs is considered too fast to allow for MRI signal acquisition, we set an out-of-plane distance (*d*) of 3 nm as the inner zone and further calculated the magnetization at *d*=5 nm in our models^36^. First, we showed that magnetic field coupling between IO NPs does not lead to a higher stray field or field gradient because the compact geometry partially neutralizes the magnetic field coupling effect (Supplementary Fig. 10). However, the regularized magnetic field coupling between proximal IO NPs offers a greatly enhanced field inhomogeneity, which could explain the increased *r*_2_ values of clusters over those of single particles (Fig. 3g,h and Supplementary Fig. 11). To better represent the field inhomogeneity around the IO clusters, we further conducted LLG simulations of multiple-particle models which imply obvious differences in field asymmetry around the cluster models C1, C2, and C3 (Supplementary Fig. 12). The 3D field inhomogeneity around the cluster models C1, C2, and C3 are visualized in Supplementary Movie 1.

We further resolved a number of models with different model settings (C1, C2, C3, and C3'), such as the relative orientation of adjacent particles to external magnetic field (Supplementary Figs 13 and 14). The strongest stray field gradient of clusters C3 or C3' is in agreement with the obtained highest *r*_2_ value of IO cluster C3. Moreover, these results indicate that the induced local magnetic field inhomogeneity by adjacent particles is greatly dependent on the orientation of the particles relative to the direction of the external magnetic field. The fundamental basis lies in the phenomena of proton diffusion and dephasing around MNPs, which is considerable as an averaged behaviour from a batch of protons (Supplementary Fig. 15). *T*_2_ relaxivity is considered independent on the size of particles in SDR, whereas our results further indicate that nanoscopic magnetic field inhomogeneity plays an important role in *T*_2_ relaxation enhancement. The abstraction of static stray field strength and diffusive field gradient may be a way to propagate the classic proton dephasing mechanism in both MAR and SDR.

### Clusters of iron oxide cubes and plates

We also employed IO cubes and IO plates as examples to study the magnetic field inhomogeneity in *T*_2_ relaxation enhancement of anisotropically shaped magnetic nanostructures. The IO cubes with side length of 12 nm and the IO plates with side length of 12 nm and thickness of 4.8 nm have the equivalent solid volume as the IO-15 spheres (Supplementary Fig. 16). Previously, we have shown that anisotropically shaped iron oxide nanostructures exhibited higher *r*_2_ values due to the enhanced effective radius, which could generate higher levels of local field inhomogeneity^14^,^23^. In this study, we artificially combined IO-5 NPs with IO cubes (C6) or plates (C7) as building blocks for self-assembly into clusters.

**Figure 4:**
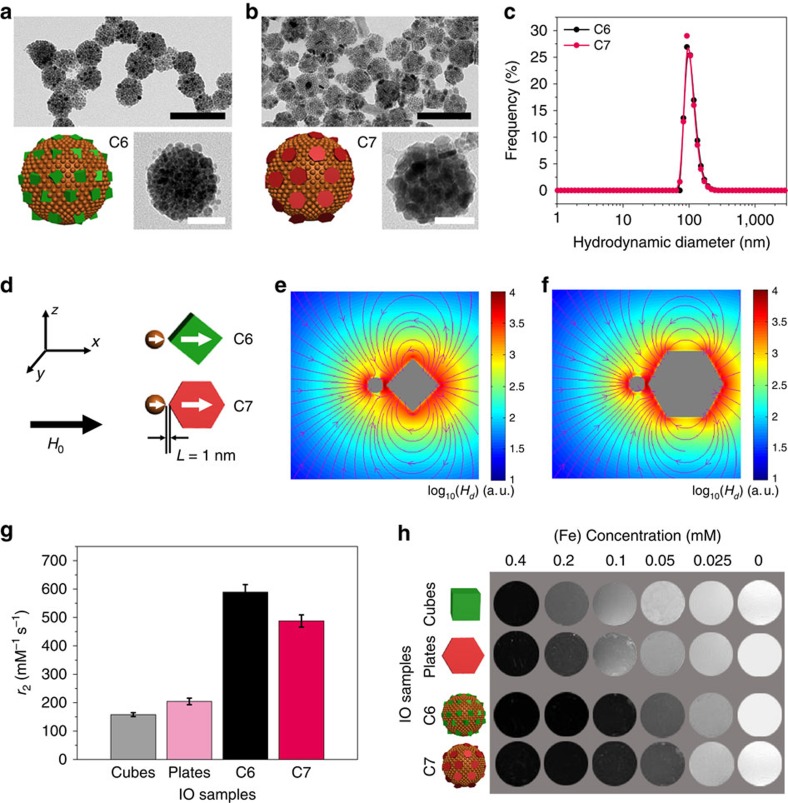
Iron oxide clusters C6 and C7. (**a**,**b**) Transmission electron microscopy (TEM) and high-resolution TEM images, as well as cartoons of iron oxide (IO) clusters C6 and C7, respectively. Scale bars, 200 nm (black), 50 nm (white). (**c**) Dynamic light scattering (DLS) measurements indicate uniform hydrodynamic diameters of IO clusters C6 and C7. (**d**–**f**) Simulation models and the calculated stray fields for the models C6 and C7, respectively. The inter-particle distance *L* is 1.0 nm. Colour bars represent log_10_(*H*_*d*_) (a.u.), where *H*_*d*_ is the calculated stray field. (**g**) Columns show *r*_2_ values of the IO clusters C6 and C7, as well as the single IO cubes and plates. Mean values±s.d.; *n*=3. (**h**) MR phantom of different IO samples show concentration- and relaxivity-dependent contrasts. Darker contrast indicates stronger *T*_2_ relaxation enhancement for each concentration. All MRI studies were conducted on a 7 T MRI scanner.

TEM and HRTEM images show that IO clusters C6 and C7 are of similar diameters of 85.7±13.9 and 91.6±15.3 nm (Fig. 4a,b). Dynamic light scattering measurements further confirmed the equivalent hydrodynamic diameters for C6 and C7 (Fig. 4c). LLG simulations based on the models of an adjacent IO-5 and IO cube (C6) or IO-5 and IO plate (C7) were performed under the same conditions as conducted on models C1–C3 (Fig. 4d–f and Supplementary Fig. 17). Because of their non-spherical shape, IO cubes and plates assemble with different orientations with respect to IO-5 NPs. Therefore, we also conducted the LLG simulations of C6 and C7 models of different configurations, C6-bx, C7-bx, C6-bz and C7-bz (Supplementary Figs 18 and 19).

The results are in good agreement with that derived from Fig. 4d–f, indicating the generality of the simulation models. MR relaxivity results showed that IO cubes and plates possess higher *r*_2_ values than IO-15 with an equivalent solid volume (Fig. 4g). The highest *r*_2_ value of 204.5±11.4 mM^−1^ s^−1^ of IO plates can be attributed to the largest effective radius among IO cubes and IO-15 NPs (Supplementary Table 1). On the contrary, we found that the *r*_2_ value of IO cluster C7 is relatively lower than that of C6, 487.7±21.5 versus 589.3±26.8 mM^−1^ s^−1^, which may be attributed to the decrease of the averaged effective radius of nanoplates following Lamer assembly in clusters (Fig. 4g). Prominently, the *r*_2_ value of C7 is still higher than that of clusters consisting of only IO plates as reported in previous literature^23^, which could be due to the artificially enhanced local field inhomogeneity by introducing IO-5 NPs in close proximity to each other. The highest *r*_2_ value of IO cluster C6 is thus attributed to the presence of spin-polarized IO cubes in clusters, which have elevated levels of field inhomogeneity compared to that of IO spheres. The imaging contrasts in MR phantom are consistent with the relaxivities of IO plates and cubes, as well as clusters C6 and C7, at a set of concentrations (Fig. 4h). Therefore, we have demonstrated that artificially enhanced local field inhomogeneity is likely a general strategy to develop high-performance *T*_2_ contrast agents based on magnetic clustering structures.

### *In vitro* study and *in vivo* MRI of liver tumours

Prior to conducting *in vivo* MRI studies, we assessed the biocompatibility of IO clusters *in vitro* using HepG2 and macrophage Raw 264.7 cells. After being incubated with IO clusters for 24 h, both HepG2 and Raw 264.7 cells had more than 90% viability at concentrations up to 100 μg Fe ml^−1^ (Supplementary Fig. 20). A histological study revealed good biocompatibility with no organ abnormality or lesion observed in mice after being treated with IO clusters (2.0 mg Fe kg^−1^ mouse body weight) for 24 h (Supplementary Fig. 21). Quantitative analysis of cellular uptake by inductively coupled plasma optical emission spectroscopy showed that RAW 264.7 cells absorbed higher levels of IO clusters than HepG2 cells (Supplementary Fig. 22), which could be caused by enhanced inflammatory responses in macrophages^46^,^47^. Due to the probable formation of a protein corona, single IO NPs also revealed aggregated features in endocytotic vesicles of RAW 264.7 cells as shown in cellular TEM images (Supplementary Fig. 23). However, cells absorbing IO clusters show higher *T*_2_ contrast and larger *T*_2_ relaxation time changes than those absorbing single IO NPs (Supplementary Fig. 24). Clinically used *T*_2_ contrast agents (for example, Resovist and Endorem) can exhibit a high degree of uncontrollable particle aggregation and therefore high *r*_2_ values (up to 200 mM^−1^ s^−1^) in biological applications^24^,^48^. Our results suggest that a synthetic approach through well-controlled self-assembly may be a general solution to obtain reliable and efficient *T*_2_ contrast agents^34^.

**Figure 5:**
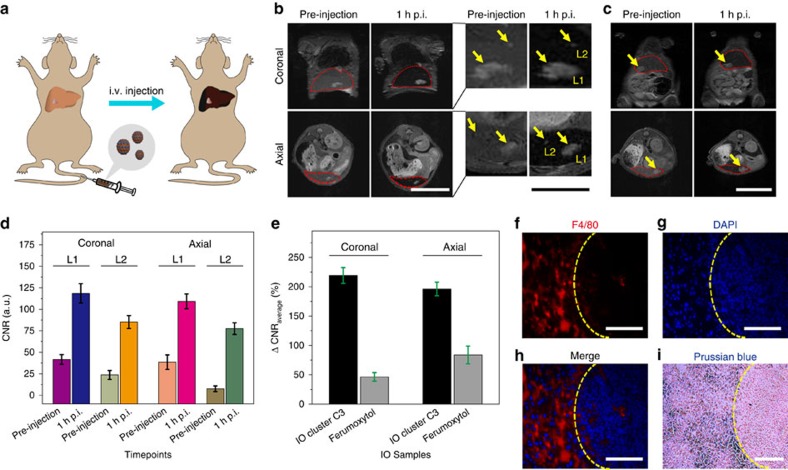
Contrast-enhanced MRI of mouse liver tumour. (**a**) Scheme of contrast-enhanced *T*_2_-weighted MRI of an orthotopic mouse liver tumour by intravenous (i.v.) injection of contrast agents. The accumulation of contrast agents in liver tissue resolves liver tumour due to the dark pseudo-contrast found in the liver tissue but not in tumour. (**b**,**c**) Coronal and axial MR images acquired at pre-injection and 1 h post injection (p.i.) of IO cluster C3 or ferumoxytol at a region of interest (ROI) in the liver (red dotted circle), respectively. The potential lesions in the liver are indicated by yellow arrows. Scale bars, 2 cm (white), 1 cm (black). (**d**) Contrast-to-noise ratio (CNR) analysis of L1 and L2 calculated by fine analysis of MR images at multiple slices using Image J. Both coronal and axial images were analysed on L1 and L2 independently and triply (*n*=3). Mean values±s.d. (**e**) Quantitative analysis of ΔCNR_average_ with respect to imaging liver tumour by IO cluster C3 and commercial ferumoxytol at coronal and axial planes. Mean values±s.d. (**f**–**h**) F4/80, DAPI staining and the merged images of the liver tissue, respectively. Yellow dotted curves indicate the boundary between normal liver tissue (left) and the liver tumour (right). Scale bars, 100 μm. (**i**) Prussian blue staining of the liver tissue indicates the decomposition of iron in normal liver tissue but not in the liver tumour. Scale bar, 100 μm.

We then conducted an *in vivo* MRI study in mouse models. Intravenously injected IO clusters are prone to accumulate in the liver through the mononuclear phagocyte system due to the fact that the mouse liver contains a large population of Kupffer cells and other macrophages^49^,^50^. Therefore, pseudo-contrast imaging is applied to enhance liver tumour contrast, as normal liver tissue show dark *T*_2_ signal owing to the accumulation of contrast agents (Fig. 5a). The orthotopic liver tumour model was established by injecting firefly luciferase-encoded HepG2 cells into the mouse liver^51^. Before MRI tests, we used bioluminescence imaging to confirm the presence of liver tumour (Supplementary Fig. 25). A preliminary *T*_2_ MRI study indicated that the maximal signal-to-noise (SNR) ratio in the liver was reached around 1 h post injection (p.i.) of IO cluster C3 (Supplementary Fig. 26). Subsequently, we acquired MR images of mice within regions of interest (ROIs) of the liver and the liver tumour both pre-injection and 1 h.p.i. of IO cluster C3, with a dose of 1.0 mg Fe kg^−1^ mouse body weight.

Compared with the surrounding liver tissue, the liver tumour is considerably devoid of Kupffer cells and thus is less likely to uptake contrast agents. Our results show that contrast-enhanced *T*_2_ images exhibit a significantly higher degree of recognition for a major liver tumour (L1) with a clear boundary to surroundings, which may greatly improve diagnostic accuracy (Fig. 5b; Supplementary Fig. 27). More interestingly, a small lesion (L2) was clearly seen at 1 h.p.i. due to the suppression of the background signal, which is barely detectable in pre-injection images (Fig. 5b).

Furthermore, we compared the feasibility of detecting liver tumours with our IO clusters to a clinically approved IO agent, ferumoxytol, with an *r*_2_ value of 103.4±6.5 mM^−1^ s^−1^ (Supplementary Fig. 28). Under similar conditions, ferumoxytol shows relatively poor *T*_2_ contrast and contrast-to-noise ratio (CNR) enhancement in detecting a liver tumour (Fig. 5c; Supplementary Fig. 29). Quantitative analysis of the CNR ratio (CNR=|SNR_tumour_−SNR_liver_|/SNR_tumour_) of L1 and L2 indicates that the CNR is significantly improved in the 1 h.p.i. images compared with the pre-injection images (Fig. 5d). The average changes of CNR (ΔCNR_average_=|CNR_post_−CNR_pre_|/CNR_pre_) show that IO C3 achieved greatly enhanced *T*_2_ contrast efficiency and sensitivity in detecting liver lesions (Fig. 5e). The presence of macrophages in liver tissue but not in liver tumour was confirmed by F4/80 staining (Fig. 5f–h), which is also indicated by the features of iron deposition from Prussian blue staining (Fig. 5i).

## Discussion

Several strategies have been employed to control the uniformity of nanomaterials at different levels, including direct synthesis of single NPs and their self-assembly into superstructures^52^,^53^,^54^,^55^. In most cases, homogeneity governs the physiochemical property of materials at the nanoscale and is far beyond aesthetic demands for materials being functional in nature^56^. For example, the optical emission of quantum dots is highly dependent on the uniformity of structural parameters including size and shape, which are particularly relevant for investigating the direct structure–activity relationship^57^,^58^. However, the magnetic field generated by MNPs is highly inhomogeneous, where the strength rapidly decreases with increasing distance away from the magnetic center. Based on proton spin–spin relaxation, it has been shown that proton diffusion around MNPs would cause the loss of phase coherence and consequently the shortening of proton *T*_2_ relaxation time^36^,^37^. Apparently, local magnetic fields induced by MNPs is the major cause for *T*_2_ relaxation enhancement of protons on MNPs. Although the related parameter *M*_s_ has been used to calculate the expected *r*_2_ values of given MNPs^10^,^11^, it was still not clear how the inhomogeneous magnetic field impacts the dynamic diffusion and dephasing of protons around MNPs. This becomes more complicated when attempting to generalize the outersphere theory of single-domain particles to multi-domain clusters.

We are specifically interested in the local magnetic field inhomogeneity caused by MNPs and its influence on proton *T*_2_ relaxation enhancement. For the first time, we have emphasized that magnetic field inhomogeneity can be resolved into static field strength and diffusive field gradient. We show that adjacent MNPs can enhance the field inhomogeneity due to the reduced symmetry of induced magnetic field by individual particles, which is applicable to explain the higher *T*_2_ contrast efficiency of polynuclear clusters over single MNPs. We further envision that proximal MNPs with different magnetizations in terms of size and shape can artificially reduce the symmetry of the induced field, thus enhancing the local field inhomogeneity. The polynuclear IO clusters were made through a well-controlled self-assembly strategy with sizes in the typical SDR range^26^. This allows us to directly compare their *T*_2_ relaxivity, focusing on the magnetic field inhomogeneity while isolating the influence of confounding parameters, such as the cluster size and water diffusivity. It is noteworthy that the *r*_2_ values of IO clusters would decrease as the size decreases to the size threshold of MAR. Our results demonstrated that the *r*_2_ value of IO clusters gradually increases, with increasing local field inhomogeneity, from small to large IO NPs and from spherical to non-spherical IO NPs. The *T*_2_ relaxivity of IO clusters is mainly attributed to the outersphere contribution by which the contribution to *T*_1_ relaxivity is negligible. On the contrary, small-sized single IO-5 NPs exhibit appreciable *T*_1_ contrast enhancement due to the considerable innersphere contribution. More importantly, the high *r*_2_/*r*_1_ ratios of these IO clusters further support the great efficacy in *T*_2_ contrast imaging (Supplementary Table 1). The higher *r*_2_ but lower *M*_s_ values of IO cluster C3 relative to that of C2 is a typical example in which local field inhomogeneity is more dependent on the diffusive field gradient rather than field strength.

Previously, Monte Carlo simulations were employed to investigate the relationship between *r*_2_ values and geometric parameters of magnetic clusters, indicating that *r*_2_ values are sensitive to the changes of particle configuration in cluster, especially in MAR^26^. However, the investigation on the magnetic field of polynuclear models consisting of particles with different geometries (for example, size and shape) is rarely done. In this study, LLG simulations were used to study the magnetization process on nanoscopic magnets, allowing us to elaborate on the magnetic field induced by single MNPs or polynuclear models. The LLG simulations were carried out on well-organized models due to the ease of computational processing and calculation. The symmetry of induced local magnetic field by the MNPs is critical as indicated by the phenomenon of proton diffusion and dephasing. Our results show that IO clusters consisting of IO-5 and IO-15 NPs exhibited higher *r*_2_ values over that of clusters consisting of IO-5 or IO-15 NPs only. The highest *r*_2_ value was obtained by replacing IO-15 NPs with IO cubes of equivalent solid volume, due to the enhanced level of local field complexity and inhomogeneity. On the other hand, the *r*_2_ value was compromised by replacing IO-15 NPs with IO plates, because the Lamer assembly of nanoplates in clusters probably reduces the magnetic field inhomogeneity. For the *in vivo* study, although IO NPs were considered to have low systemic toxicity^59^, excessive iron deposition was recently realized to induce extensive cellular damage, particularly fibrosis in the heart and liver^60^. Note that the dose (1.0 mg Fe kg^−1^) of IO clusters used in contrast-enhanced MRI of mouse liver tumour is half the dose commonly used in other studies. This may remarkably reduce the potential of systemic toxicity for pre-clinical research and potential clinical practices.

In summary, this study was devoted to the understanding of how magnetization influences *T*_2_ contrast efficiency in magnetic clusters, especially when the classic outersphere theory faces challenges. Using both experiments and theoretical simulations, we have presented that magnetic field inhomogeneity (for example, gradient and symmetry) is the major cause of *T*_2_ relaxation enhancement in IO clusters. Artificially enhanced field inhomogeneity boosts the *r*_2_ value of IO clusters consisting of IO NPs with different sizes and shapes. LLG calculations further confirmed that architectural heterogeneity with reduced symmetry between MNPs would augment the level of magnetic field inhomogeneity. These simple yet distinct results are amenable to shed light on classic outersphere theory to interpret the *T*_2_ shortening effect extended from single particles to magnetic clusters, and more importantly, suggest design considerations of polynuclear clusters for high-performance *T*_2_ MRI applications.

## Methods

### Preparation of iron oxide clusters

Amphiphilic IO NPs were synthesized using the tandem ‘grafting to' and ‘grafting from' reaction we have recently developed. In the ‘grafting to' reaction, a solution of phosphonated-PEG (PEG-P) (20 mg) and the ATRP initiator BiBEP (15 mg) in chloroform (2 ml) was added slowly into 5 ml of the IO NPs (100 nM) in chloroform. After stirring for 12 h, the samples (IO@PEG/BiBEP) were washed with a mixture of acetone and diethyl ether (2:1) and collected after centrifugation at 4,000*g* for 5 min. IO@PEG/BiBEP was dispersed in 4 ml of DMF for further uses. In the ‘grafting from' reaction, MMA (0.4 ml) and IO@PEG/BiBEP (50 nM) were mixed in DMF (2 ml). After being degassed for 30 min by N_2_, CuBr (6 mg) and ME_6_TREN (35 mg) were added, and the reaction solution was kept in a water bath at 40 °C for 12 h. After being purified by magnetic separation five times, the IO NPs coated with PEG and PMMA (IO@PEG/PMMA) were stored in chloroform. To prepare IO cluster C3 for example, 60 μl (850 nM) of IO-5@PEG/PMMA and 40 μl (50 nM) of IO-15@PEG/PMMA in chloroform were mixed with 900 μl (0.05 mg ml^−1^) of SDS in water and emulsified for ∼5 min by pulsed sonication (100 W and 22.5 kHz, XL-2000 series). The emulsion was then stirred at room temperature for 10 h to evaporate the organic solvent. Other IO cluster samples were prepared by the similar method but with different feed molar ratios and starting IO NPs.

### MR phantom study and relaxivity measurements

MRI tests were conducted on a Bruker 7 T scanner (Pharmascan) equipped with a small animal-specific body coil. Samples of IO clusters or single IO NPs containing 1% of agarose gel were prepared in tubes with different concentrations of 0.4, 0.2, 0.1, 0.05 and 0.025 mM with respect to iron mass. The MR images were obtained using spin echo sequence with parameters as follows: repetition time=3,000 ms, echo time=10, 20, 30, 40, 50, 60, 70, 80, 90, 100, 110, 120, 130, 140, 150, 160 ms, matrix=256 × 256, field of view=40 × 40 mm^2^, slice thickness=3.00 mm. The *T*_2_ relaxation times were calculated by fitting these multiple spin echo images.

### LLG simulation and calculation

The micromagnetic simulations were carried out by solving the LLG equation:


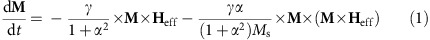


In this equation, 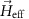 is the effective field and *M*_s_ is the saturation magnetization. We set the gyromagnetic ratio *γ*=1.78 × 10^7^ s^−1^ T^−1^, the damping constant *α*=1, the exchange stiffness constant as 1.0e-11 J m^−1^, the Gilbert damping constant as 1.0, and unit cell dimensions of 1 × 1 × 1 nm^3^. The saturation magnetization *M*_s_ was determined from the experimental data. The effective field contains contributions from the static external field, the magnetostatic field, the anisotropy field, and the exchange field. The magnetostatic field is calculated after the NP reaches a stable magnetization state under a static external field of 7 T. The stray field gradient is then calculated by the equation:


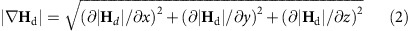


The magnetic field data from LLG was tabulated with Matlab to show the magnetic field distribution outside the single IO NPs or IO cluster models.

### Mouse orthotopic tumour model

HepG2 cells were obtained from the American Type Culture Collection (ATCC) and grown in DMEM cell culture medium. Cells were collected with 1 mM EDTA in calcium–magnesium-free phosphate-buffered saline (PBS) and resuspended in serum-free culture medium. Matrigel was used to avoid diffusion out of the injected cells. Prior to injection, the cells (5 × 10^5^) were mixed with sterile Matrigel (BD Biosciences) at a 1:1 ratio and kept on ice until injection. The nude mouse was anaesthetized with isoflurane inhalation. The abdomen was sterilized with iodine and alcohol swabs and a small (0.5–1 cm) incision was made in the skin above the liver. The syringe was gently advanced into the liver and the tumour cell mixture was injected. The abdominal wall was sutured using a 6.0 vicryl absorbable suture and a 6.0 prolene nonabsorbable suture for the skin layer.

### *In vivo* study of contrast-enhanced *T*_2_-weighted MRI

All animal studies were conducted following a protocol approved by the National Institutes of Health Clinical Center Animal Care and Use Committee (NIH CC/ACUC). Mice were anaesthetized by isoflurane (1.0–2.0%) in oxygen and placed in an animal-specific body coil for MRI data acquisition. Mice were kept warm by circulating warm water (37 °C) and were placed in a stretched prone position with a respiratory sensor during the experiments. *T*_2_-weighted images were acquired at pre-injection and post injection (p.i.) of contrast agents intravenously on both coronal and axial planes, focusing on the ROI of mouse liver. Multi-slice multi-echo sequence was employed to acquire images using parameters as follows: repetition time=2,000 ms, echo time=30 ms, flip angle=180°, matrix size=256 × 256, field of view=40 × 40 mm^2^, slices=16, slice thickness=1 mm. A MR compatible small animal respiratory gating device was utilized to reduce the artefacts caused by respiration. MR images were analysed by measuring signal intensity in ROI with NIH supported software Image J. SNR was calculated according to the methods provided by the National Electrical Manufacturers Association (NEMA) standards publication (MS 6-2008, R2014) on single-image measurement procedure for SNR. The following equations were used to deduce CNR: SNR=SI_mean_/SD_noise_; ΔSNR=|SNR_post_−SNR_pre_|/SNR_pre_; CNR=|SNR_tumour_−SNR_liver_|/SNR_tumour_; ΔCNR=|CNR_post_−CNR_pre_|/CNR_pre_.

### Data availability

The authors declare that the data supporting the findings of this study are available within the article and its Supplementary Information Files or from the corresponding author on reasonable request.

## Additional information

**How to cite this article:** Zhou, Z *et al*. Artificial local magnetic field inhomogeneity enhances *T*_2_ relaxivity. *Nat. Commun.* **8**, 15468 doi: 10.1038/ncomms15468 (2017).

**Publisher's note**: Springer Nature remains neutral with regard to jurisdictional claims in published maps and institutional affiliations.

## Supplementary Material

Supplementary Information Supplementary Figures, Supplementary Table, Supplementary Methods and Supplementary References.

Supplementary Movie 1 3D-view of the field inhomogeneity induced by IO cluster models C1, C2 and C3 with an external magnetic field along +x direction. The movie is a side view of stray field gradient of the spinning multiple-particle models C1, C2, and C3.

Peer Review File

## References

[b1] Reiss G. & Hutten A. Magnetic nanoparticles: applications beyond data storage. Nat. Mater. 4, 725–726 (2005). 16195762 10.1038/nmat1494

[b2] Lee H., Shin T.-H., Cheon J. & Weissleder R. Recent developments in magnetic diagnostic systems. Chem. Rev. 115, 10690–10724 (2015). 26258867 10.1021/cr500698d PMC5791529

[b3] Perez J. M., Josephson L., O'Loughlin T., Hogemann D. & Weissleder R. Magnetic relaxation switches capable of sensing molecular interactions. Nat. Biotechnol. 20, 816–820 (2002). 12134166 10.1038/nbt720

[b4] Laurent S. . Magnetic iron oxide nanoparticles: synthesis, stabilization, vectorization, physicochemical characterizations, and biological applications. Chem. Rev. 108, 2064–2110 (2008). 18543879 10.1021/cr068445e

[b5] Lee N. . Iron oxide based nanoparticles for multimodal imaging and magnetoresponsive therapy. Chem. Rev. 115, 10637–10689 (2015). 26250431 10.1021/acs.chemrev.5b00112

[b6] Liang Z. P. & Lauterbur P. C. Principles of Magnetic Resonance Imaging: a Signal Processing Perspective Wiley-IEEE Press (1999).

[b7] Gallo J., Long N. J. & Aboagye E. O. Magnetic nanoparticles as contrast agents in the diagnosis and treatment of cancer. Chem. Soc. Rev. 42, 7816–7833 (2013). 23788179 10.1039/c3cs60149h

[b8] Lee N. & Hyeon T. Designed synthesis of uniformly sized iron oxide nanoparticles for efficient magnetic resonance imaging contrast agents. Chem. Soc. Rev. 41, 2575–2589 (2012). 22138852 10.1039/c1cs15248c

[b9] Shin T.-H., Choi Y., Kim S. & Cheon J. Recent advances in magnetic nanoparticle-based multi-modal imaging. Chem. Soc. Rev. 44, 4501–4516 (2015). 25652670 10.1039/c4cs00345d

[b10] Hwang L. P. & Freed J. H. Dynamic effects of pair correlation functions on spin relaxation by translational diffusion in liquids. J. Chem. Phys. 63, 4017–4025 (1975).

[b11] Freed J. H. Dynamic effects of pair correlation functions on spin relaxation by translational diffusion in liquids. II. Finite jumps and independent T_1_ processes. J. Chem. Phys. 68, 4034 (1978).

[b12] Jun Y. W. . Nanoscale size effect of magnetic nanocrystals and their utilization for cancer diagnosis via magnetic resonance imaging. J. Am. Chem. Soc. 127, 5732–5733 (2005). 15839639 10.1021/ja0422155

[b13] Tromsdorf U. I. . Size and surface effects on the MRI relaxivity of manganese ferrite nanoparticle contrast agents. Nano Lett. 7, 2422–2427 (2007). 17658761 10.1021/nl071099b

[b14] Zhao Z. . Octapod iron oxide nanoparticles as high-performance *T*_2_ contrast agents for magnetic resonance imaging. Nat. Commun. 4, 2266 (2013). 23903002 10.1038/ncomms3266

[b15] Lee N. . Water-dispersible ferrimagnetic iron oxide nanocubes with extremely high r_2_ relaxivity for highly sensitive *in vivo* MRI of tumors. Nano Lett. 12, 3127–3131 (2012). 22575047 10.1021/nl3010308

[b16] Sharma V. K., Alipour A., Soran-Erdem Z., Aykut Z. G. & Demir H. V. Highly monodisperse low-magnetization magnetite nanocubes as simultaneous T1-T2 MRI contrast agents. Nanoscale 7, 10519–10526 (2015). 26010145 10.1039/c5nr00752f

[b17] Wetterskog E., Tai C.-W., Grins J., Bergström L. & Salazar-Alvarez G. Anomalous magnetic properties of nanoparticles arising from defect structures: topotaxial oxidation of Fe1–xO|Fe3−δO4 core|shell nanocubes to single-phase particles. ACS Nano 7, 7132–7144 (2013). 23899269 10.1021/nn402487q

[b18] Voros E. . TPA immobilization on iron oxide nanocubes and localized magnetic hyperthermia accelerate blood clot lysis. Adv. Funct. Mater. 25, 1709–1718 (2015).

[b19] Lee J. H. . Artificially engineered magnetic nanoparticles for ultra-sensitive molecular imaging. Nat. Med. 13, 95–99 (2007). 17187073 10.1038/nm1467

[b20] Jang J. T. . Critical enhancements of MRI contrast and hyperthermic effects by dopant-controlled magnetic nanoparticles. Angew. Chem. Int. Ed. 48, 1234–1238 (2009). 10.1002/anie.200805149 19137514

[b21] Liu Y. . Deep photoacoustic/luminescence/magnetic resonance multimodal imaging in living subjects using high-efficiency upconversion nanocomposites. Adv. Mater. 28, 6411–6419 (2016). 27185066 10.1002/adma.201506460

[b22] Zhou Z. . A synergistically enhanced T_1_-T_2_ dual-modal contrast agent. Adv. Mater. 24, 6223–6228 (2012). 22972529 10.1002/adma.201203169 PMC3634350

[b23] Zhou Z. . Interplay between longitudinal and transverse contrasts in Fe_3_O_4_ nanoplates with (111) exposed surfaces. ACS Nano 8, 7976–7985 (2014). 25093532 10.1021/nn5038652 PMC4568839

[b24] Pöselt E. . Relaxivity optimization of a PEGylated iron-oxide-based negative magnetic resonance contrast agent for T2-weighted spin–echo imaging. ACS Nano 6, 1619–1624 (2012). 22276942 10.1021/nn204591r

[b25] Tong S., Hou S., Zheng Z., Zhou J. & Bao G. Coating optimization of superparamagnetic iron oxide nanoparticles for high T_2_ relaxivity. Nano Lett. 10, 4607–4613 (2010). 20939602 10.1021/nl102623x PMC3170660

[b26] Matsumoto Y. & Jasanoff A. T2 relaxation induced by clusters of superparamagnetic nanoparticles: Monte Carlo simulations. Magn. Reson. Imaging 26, 994–998 (2008). 18479873 10.1016/j.mri.2008.01.039

[b27] Shao H. . Magnetic nanoparticles and microNMR for diagnostic applications. Theranostics 2, 55–65 (2012). 22272219 10.7150/thno.3465 PMC3263516

[b28] Shapiro M. G. . Directed evolution of a magnetic resonance imaging contrast agent for noninvasive imaging of dopamine. Nat. Biotechnol. 28, 264–270 (2010). 20190737 10.1038/nbt.1609 PMC3073400

[b29] Haun J. B. . Micro-NMR for rapid molecular analysis of human tumor samples. Sci. Transl. Med. 3, 71ra16 (2011). 10.1126/scitranslmed.3002048 PMC3086073 21346169

[b30] Pothayee N. . Magnetic nanoclusters with hydrophilic spacing for dual drug delivery and sensitive magnetic resonance imaging. J. Mater. Chem. B 1, 1142–1149 (2013). 10.1039/C2TB00275B PMC4199085 25328679

[b31] Balasubramaniam S. . Toward design of magnetic nanoparticle clusters stabilized by biocompatible diblock copolymers for T2-weighted MRI contrast. Langmuir 30, 1580–1587 (2014). 24479874 10.1021/la403591z

[b32] Mehdizadeh Taheri S. . Self-assembly of smallest magnetic particles. Proc. Natl Acad. Sci. USA 112, 14484–14489 (2015). 26554000 10.1073/pnas.1511443112 PMC4664362

[b33] Mikhaylov G. . Ferri-liposomes as an MRI-visible drug-delivery system for targeting tumours and their microenvironment. Nat. Nanotechnol. 6, 594–602 (2011). 21822252 10.1038/nnano.2011.112

[b34] Hickey R. J., Haynes A. S., Kikkawa J. M. & Park S.-J. Controlling the self-assembly structure of magnetic nanoparticles and amphiphilic block-copolymers: from micelles to vesicles. J. Am. Chem. Soc. 133, 1517–1525 (2011). 21208004 10.1021/ja1090113

[b35] Vuong Q. L., Berret J. F., Fresnais J., Gossuin Y. & Sandre O. A universal scaling law to predict the efficiency of magnetic nanoparticles as MRI T_2_-contrast agents. Adv. Healthc. Mater. 1, 502–512 (2012). 23184784 10.1002/adhm.201200078

[b36] Gillis P., Moiny F. & Brooks R. A. On T2-shortening by strongly magnetized spheres: a partial refocusing model. Magnet. Reson. Med. 47, 257–263 (2002). 10.1002/mrm.10059 11810668

[b37] Brooks R. A., Moiny F. & Gillis P. On T2-shortening by weakly magnetized particles: the chemical exchange model. Magnet. Reson. Med. 45, 1014–1020 (2001). 10.1002/mrm.1135 11378879

[b38] Carroll M. R. J. . Experimental validation of proton transverse relaxivity models for superparamagnetic nanoparticle MRI contrast agents. Nanotechnology 21, 035103 (2010). 19966406 10.1088/0957-4484/21/3/035103

[b39] Bowen C. V., Zhang X., Saab G., Gareau P. J. & Rutt B. K. Application of the static dephasing regime theory to superparamagnetic iron-oxide loaded cells. Magnet. Reson. Med. 48, 52–61 (2002). 10.1002/mrm.10192 12111931

[b40] Song J. . Ultrasmall gold nanorod vesicles with enhanced tumor accumulation and fast excretion from the body for cancer therapy. Adv. Mater. 27, 4910–4917 (2015). 26198622 10.1002/adma.201502486

[b41] Ovejero J. G. . Effects of inter- and intra-aggregate magnetic dipolar interactions on the magnetic heating efficiency of iron oxide nanoparticles. Phys. Chem. Chem. Phys. 18, 10954–10963 (2016). 27041536 10.1039/c6cp00468g

[b42] Zhang S. & Zhang S. S. L. Generalization of the Landau-Lifshitz-Gilbert equation for conducting ferromagnets. Phys. Rev. Lett. 102, 086601 (2009). 19257763 10.1103/PhysRevLett.102.086601

[b43] Nembach H. T., Shaw J. M., Boone C. T. & Silva T. J. Mode- and size-dependent Landau-Lifshitz damping in magnetic nanostructures: evidence for nonlocal damping. Phys. Rev. Lett. 110, 117201 (2013). 25166568 10.1103/PhysRevLett.110.117201

[b44] Cimrák I. A survey on the numerics and computations for the Landau-Lifshitz equation of micromagnetism. Arch. Comput. Method. E 15, 1–37 (2007).

[b45] Scheinfein M. R. . Micromagnetics of domain walls at surfaces. Phys. Rev. B 43, 3395–3422 (1991). 10.1103/physrevb.43.3395 9997652

[b46] Weissleder R., Nahrendorf M. & Pittet M. J. Imaging macrophages with nanoparticles. Nat. Mater. 13, 125–138 (2014). 24452356 10.1038/nmat3780

[b47] Miller M. A. . Tumour-associated macrophages act as a slow-release reservoir of nano-therapeutic Pt(IV) pro-drug. Nat. Commun. 6, 8692 (2015). 26503691 10.1038/ncomms9692 PMC4711745

[b48] Bruns O. T. . Real-time magnetic resonance imaging and quantification of lipoprotein metabolism *in vivo* using nanocrystals. Nat. Nanotechnol. 4, 193–201 (2009). 19265850 10.1038/nnano.2008.405

[b49] Chow A., Brown B. D. & Merad M. Studying the mononuclear phagocyte system in the molecular age. Nat. Rev. Immunol. 11, 788–798 (2011). 22025056 10.1038/nri3087

[b50] Geissmann F., Gordon S., Hume D. A., Mowat A. M. & Randolph G. J. Unravelling mononuclear phagocyte heterogeneity. Nat. Rev. Immunol. 10, 453–460 (2010). 20467425 10.1038/nri2784 PMC3032581

[b51] Rashidi B. . An orthotopic mouse model of remetastasis of human colon cancer liver metastasis. Clin. Cancer Res. 6, 2556–2561 (2000). 10873112

[b52] Singh G. . Self-assembly of magnetite nanocubes into helical superstructures. Science 345, 1149–1153 (2014). 25061133 10.1126/science.1254132

[b53] Wang T. . Self-assembled colloidal superparticles from nanorods. Science 338, 358–363 (2012). 23087242 10.1126/science.1224221

[b54] Song J. . Plasmonic vesicles of amphiphilic gold nanocrystals: self-assembly and external-stimuli-triggered destruction. J. Am. Chem. Soc. 133, 10760–10763 (2011). 21699155 10.1021/ja204387w

[b55] Nie Z., Petukhova A. & Kumacheva E. Properties and emerging applications of self-assembled structures made from inorganic nanoparticles. Nat. Nanotechnol. 5, 15–25 (2010). 20032986 10.1038/nnano.2009.453

[b56] Xia Y. Editorial: are we entering the nano era? Angew. Chem. Int. Ed. 53, 12268–12271 (2014). 10.1002/anie.201406740 25256914

[b57] Peng X. . Shape control of CdSe nanocrystals. Nature 404, 59–61 (2000). 10716439 10.1038/35003535

[b58] Chen O. . Compact high-quality CdSe–CdS core–shell nanocrystals with narrow emission linewidths and suppressed blinking. Nat. Mater. 12, 445–451 (2013). 23377294 10.1038/nmat3539 PMC3677691

[b59] Liu G., Gao J., Ai H. & Chen X. Applications and potential toxicity of magnetic iron oxide nanoparticles. Small 9, 1533–1545 (2013). 23019129 10.1002/smll.201201531

[b60] Wang M. . Combined histological and hematological assessment of iron-induced organ damage in a gerbil model of iron overload. Am. J. Transl. Res. 7, 385–392 (2015). 25901205 PMC4399101

