# Supplementary material for: Artificial local magnetic field inhomogeneity enhances T2 relaxivity
Source: Nat Commun. 2017 May 18;8:15468. doi: 10.1038/ncomms15468 (PMC5454366; doi:10.1038/ncomms15468)
Supplement: Supplementary Information — Supplementary Figures, Supplementary Table, Supplementary Methods and Supplementary References. [file ncomms15468-s1.pdf]

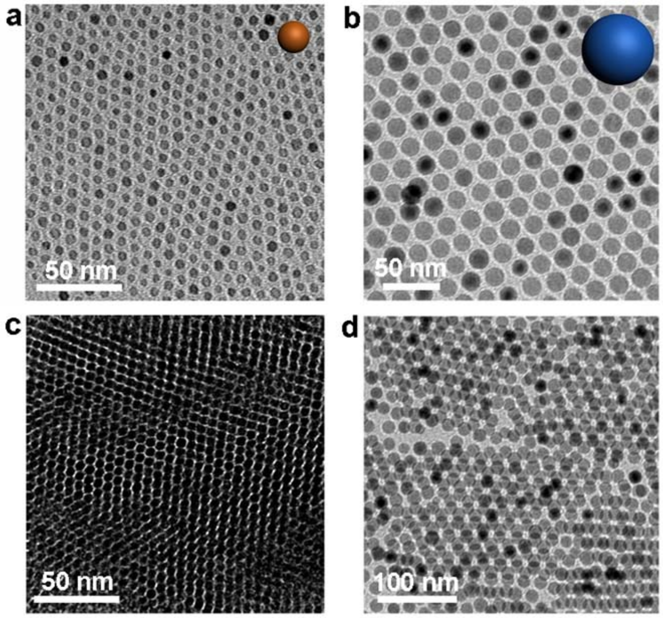

Supplementary Fig. 1 | TEM images of the single IO NPs. The single layer (a,b) and multi-layer (c,d) self-assembling onto copper grid indicates the uniform diameters of IO NPs. The averaged diameters are 5.2 and 15.1 nm with standard deviations of 4.4% and 2.9% for IO-5 and IO-15 NPs, respectively.

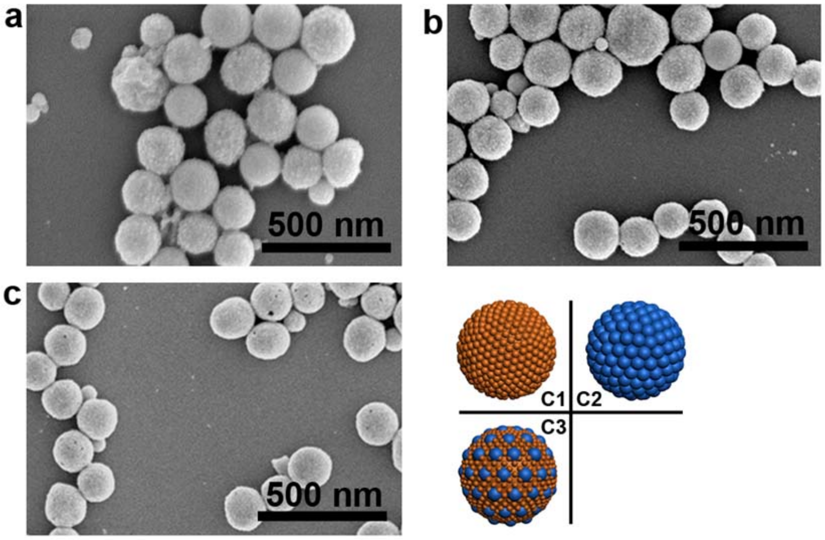

Supplementary Fig. 2 | SEM images of the IO clusters C1-C3. (a-c) SEM images of IO clusters C1-C3, respectively. The samples were coated with a layer of gold of a thickness of 3 nm before testing.

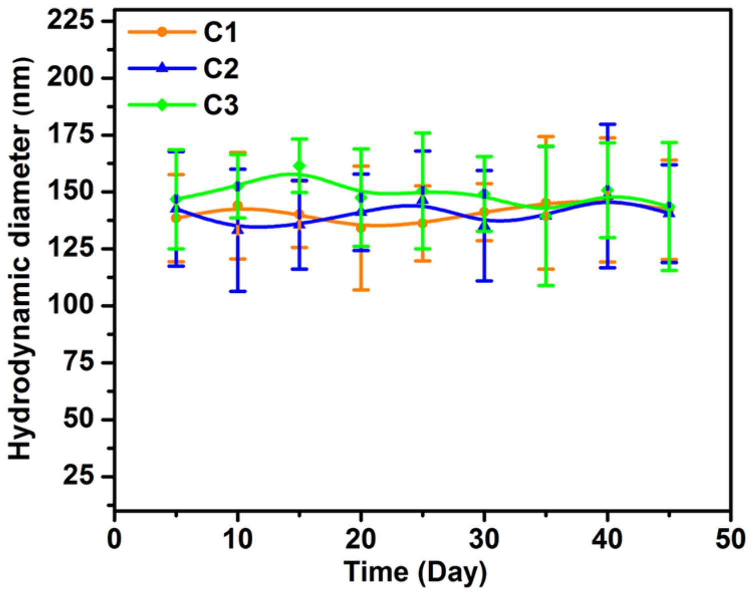

Supplementary Fig. 3 | DLS measurements of the IO clusters C1-C3. Hydrodynamic diameters of IO clusters C1-C3 in aqueous solution through a period of 45 days after preparation, indicating good colloidal stability. Errors are mean values ± s.d.; $n = 3$.

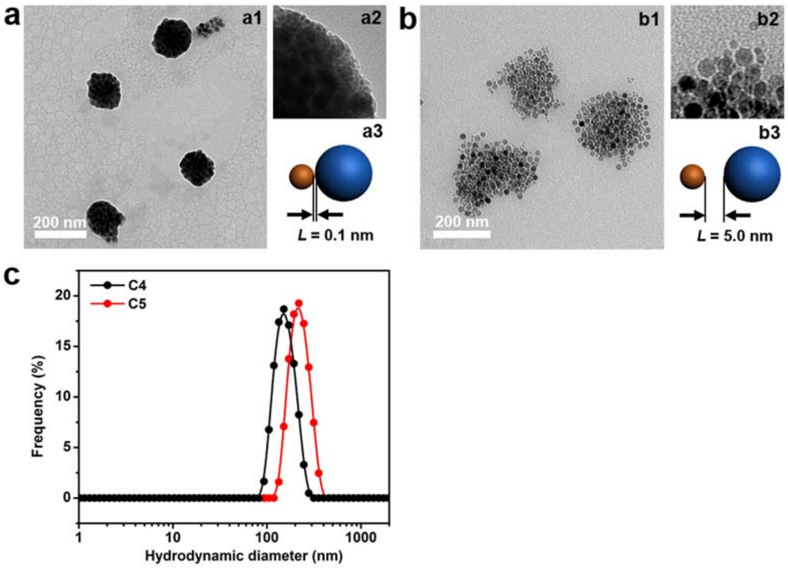

Supplementary Fig. 4 | TEM images and DLS analysis of the IO clusters C4 and C5. (a) C4 and (b) C5 composed of IO-5 and IO-15 NPs. (a1, b1) TEM and (a2, b2) HRTEM images and (a3, b3) cartoons show the different inter-particle distances (*L*) of about 0.1 and 5.0 nm for C4 and C5 respectively. (c) DLS measurement of IO clusters C4 and C5 dispersed in water.

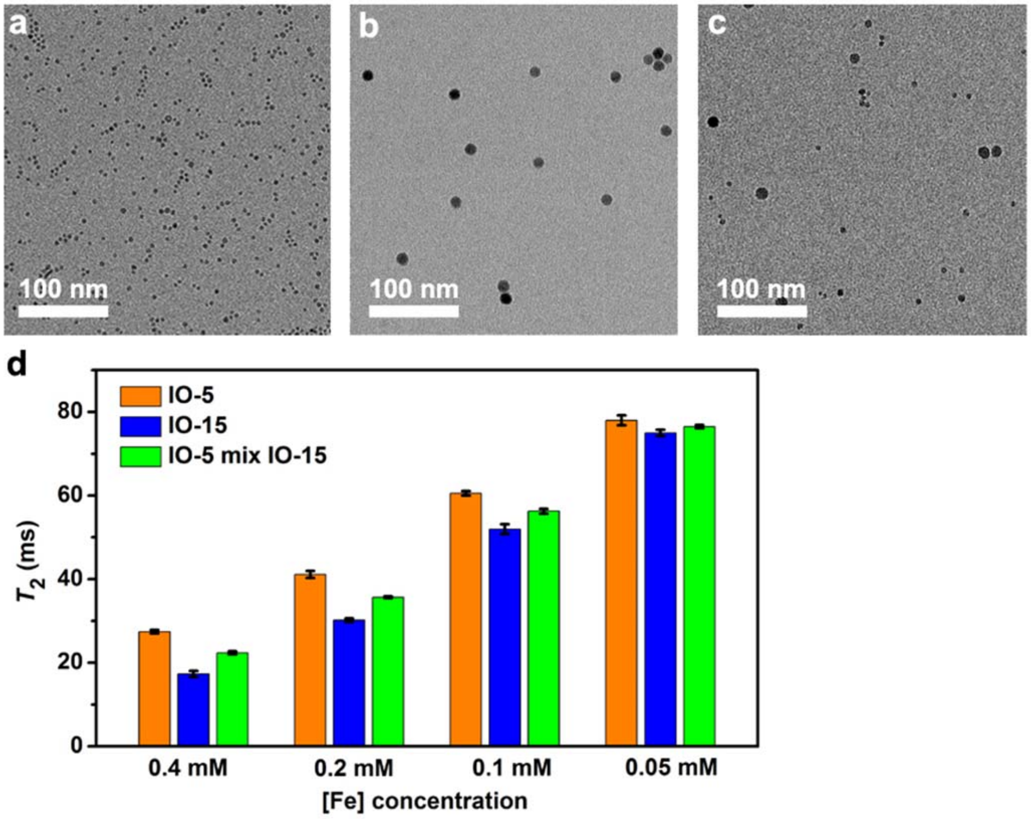

Supplementary Fig. 5 | TEM images and T_2 relaxation times of the single IO NPs. The single IO NPs coated with meso-dimercaptosuccinic acid (DMSA) molecules and dispersed in water: (a) IO-5, (b) IO-15, and (c) IO-5 plus IO-15 NPs mixed in a ratio of 1:1 with respect to iron mass. (d) The T_2 relaxation times of the IO-5, IO-15, and IO-5 mix IO-15 samples at different concentrations, indicating an averaged relaxation enhancement effect for the sample with mixed component compared with samples with individual component. Errors are mean values ± s.d.; $n = 3$.

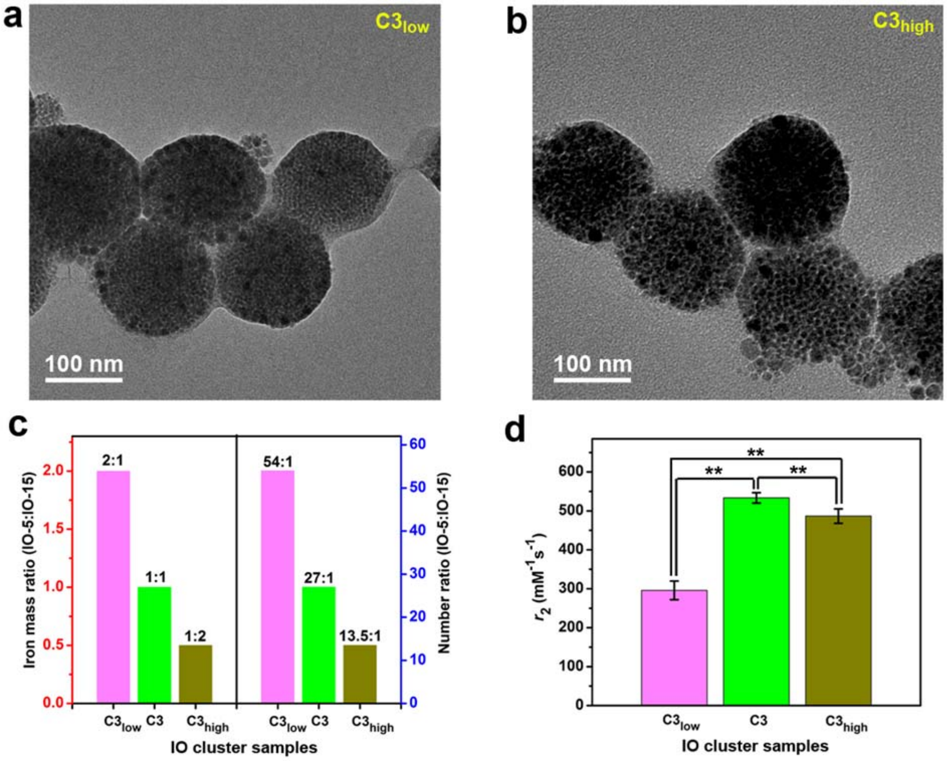

Supplementary Fig. 6 | TEM images and relaxivities of IO clusters C3$_{low}$ and C3$_{high}$ samples. (a, b) TEM images of IO cluster C3$_{low}$ and C3$_{high}$ samples, respectively. (c) The iron mass ratios are 2:1, 1:1, and 1:2 with respect to IO-5 to IO-15 NPs in IO cluster C3$_{low}$, C3, and C3$_{high}$ samples, while the number ratios are 54:1, 27:1, and 13.5:1, respectively. (d) Comparison of r_2 values for IO cluster C3$_{low}$, C3, and C3$_{high}$ samples (n = 3; **$p < 0.01$).

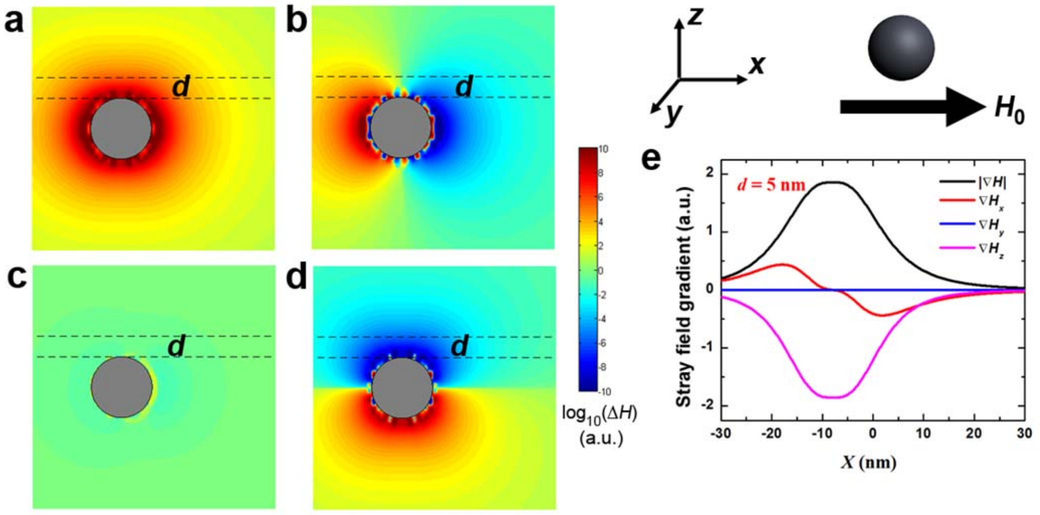

Supplementary Fig. 7 | Pilot modeling of a single IO NP. A IO NP was placed under an external magnetic field along the +*x* direction. (a) Integrated stray field gradient ($|\Delta H|$) for all directions. (b-d) Stray field gradient along *x*, *y*, and *z* directions, respectively. $\Delta H_x = \frac{\partial H}{\partial x}$, $\Delta H_y = \frac{\partial H}{\partial y}$, $\Delta H_z = \frac{\partial H}{\partial z}$. Color bars represent $\log_{10}(\Delta H)$ (a.u.), where H is the calculated stray field at *x*, *y*, or *z* direction. (e) Calculated stray field gradient for the figures a-d. The results indicated that the stray field gradient at the plane perpendicular to *z* direction (*d* = 5) mainly comes from components in the *x* and *z* directions. The positive or negative trends for ΔH_x, ΔH_y, and ΔH_z indicate the increase or decrease of stray field gradient at these directions, respectively.

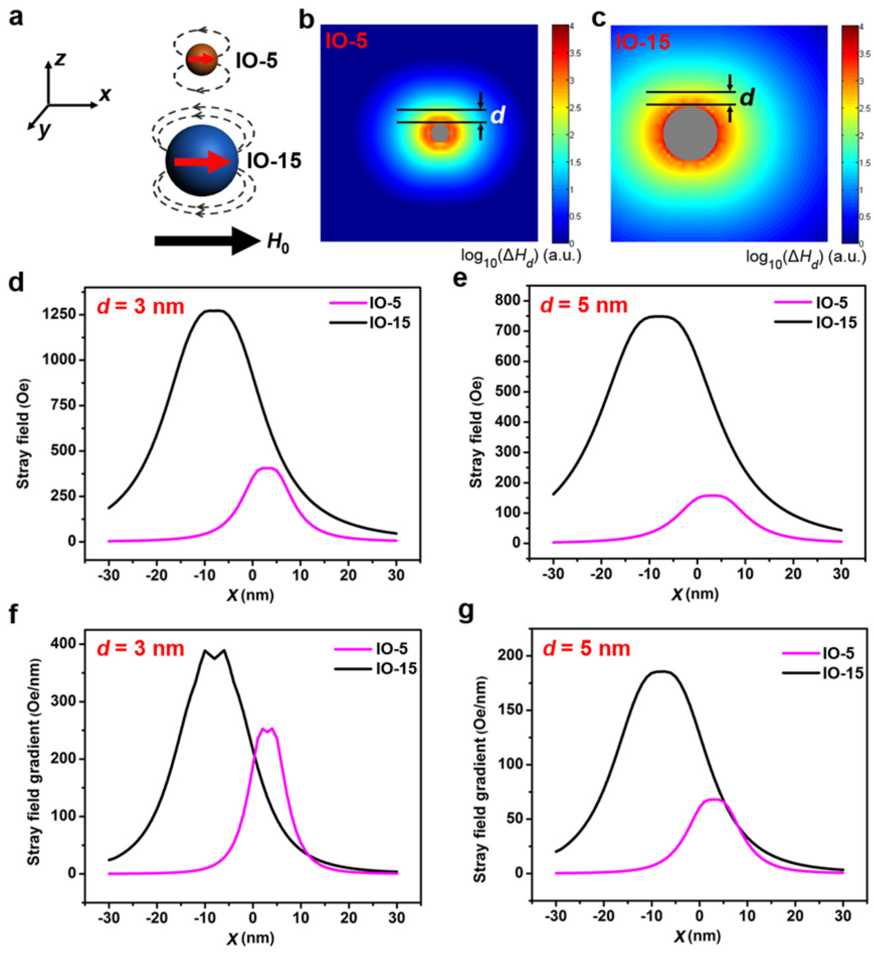

Supplementary Fig. 8 | Simulation results of single IO-5 and IO-15 NPs. (a) Cartoon shows the models for calculating the stray field and field gradient of single IO NPs (IO-5 and IO-15). An external magnetic field (7 T) is applied along the +*x* direction. (b,c) Simulated results of stray field gradient for IO-5 and IO-15 NPs. Color bars represent $\log_{10}(\Delta H_d)$ (a.u.), where H_d is the calculated stray field. The stray field (d,e) and field gradient (f,g) of IO-5 and IO-15 NPs calculated on the *xy* plane with different distances of d (3 and 5 nm) from the top of IO NPs.

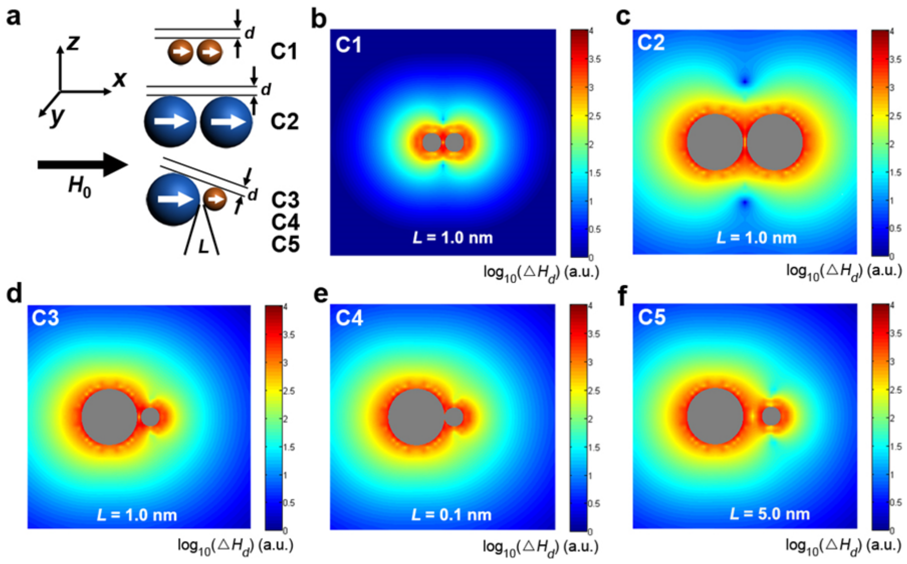

Supplementary Fig. 9 | Additional simulation results of C1-C5. The stray field gradient for models C1-C5. (a) Cartoon models show an external magnetic field (7 T) is applied along the +*x* direction for simulation and calculation. (b-f) Simulated stray field gradient for models C1-C5. Color bars represent $\log_{10}(\Delta H_d)$ (a.u.), where H_d is the calculated stray field.

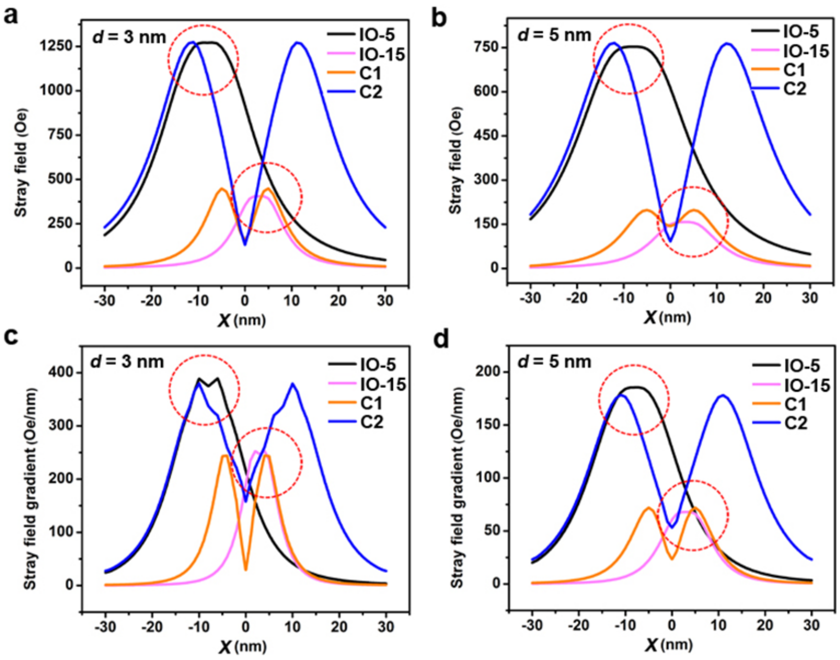

Supplementary Fig. 10 | Comparison of IO-5, IO-15, C1, and C2 models. Comparison of (a, b) stray field and (c, d) stray field gradient generated by IO-5, IO-15, C1, and C2 models at distances (*d*) of 3 and 5 nm. The results indicate that the inter-particle magnetic field coupling does not lead to higher magnetic field as compared to those of IO-5 to C1 or IO-15 to C2 (highlighted by red dotted circles).

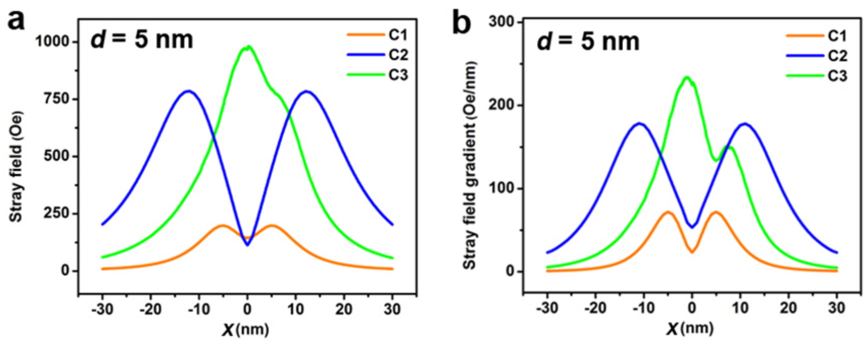

Supplementary Fig. 11 | Additional simulation results of C1-C3. The simulation results of stray field and stray field gradient of C1-C3 at a distance (*d*) of 5 nm when an external magnetic field along the +*x* direction is applied.

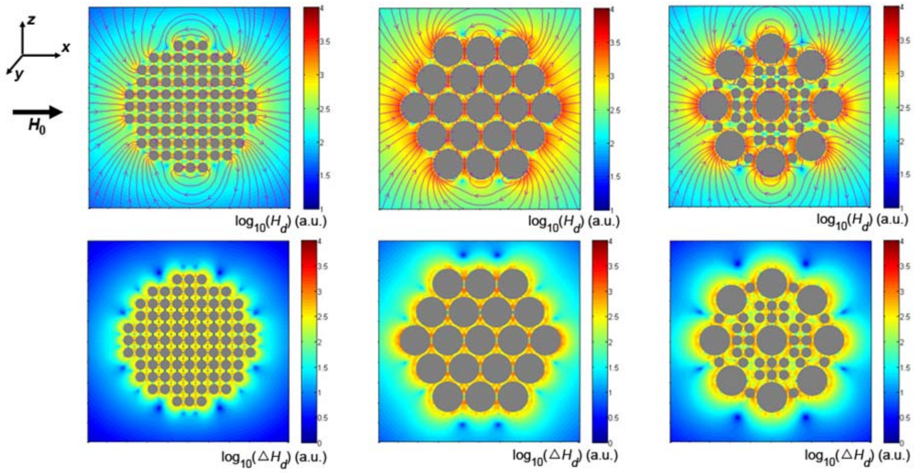

Supplementary Fig. 12 | LLG simulation results of the multiple-particle models. The external magnetic field is along +*x* direction. The stray field (upper row) and stray field gradient (lower row) of the IO cluster models C1, C2, and C3 show obvious differences in field inhomogeneity around the IO cluster models, which are in good agreement with that derived from the two-particle models. Color bars represent $\log_{10}(H_d)$ (a.u.) for the upper row and $\log_{10}(\Delta H_d)$ (a.u.) for the lower row, where H_d is the calculated stray field.

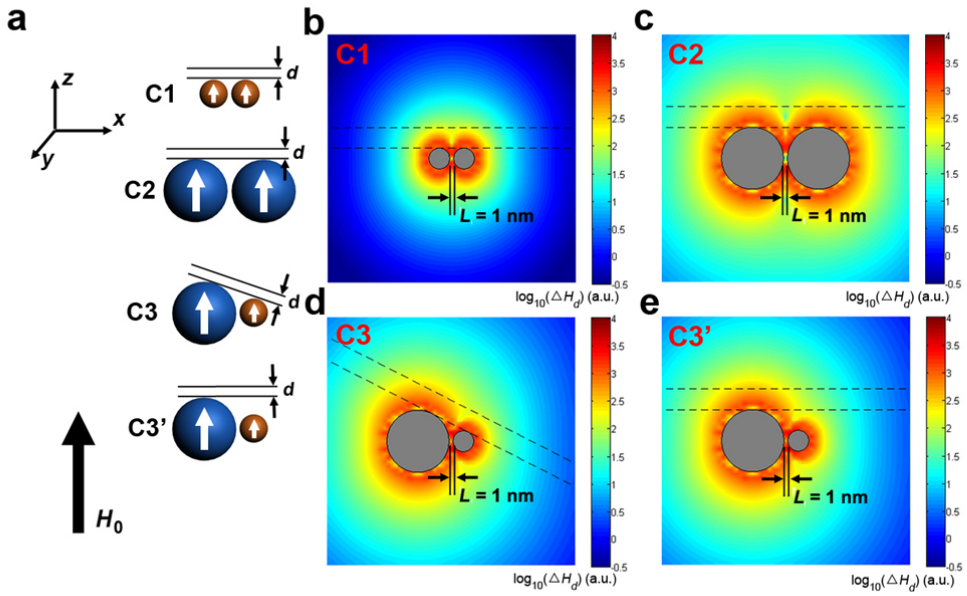

Supplementary Fig. 13 | Additional simulation results for C1, C2, C3, and C3'. (a) Cartoon models and (b-e) simulated stray field gradient for IO clusters (C1, C2, C3 and C3') with an external magnetic field (7 T) applied along the +z direction. The inter-particle distance (*L*) is 1 nm for all the models. Color bars represent $\log_{10}(\Delta H_d)$ (a.u.), where H_d is the calculated stray field.

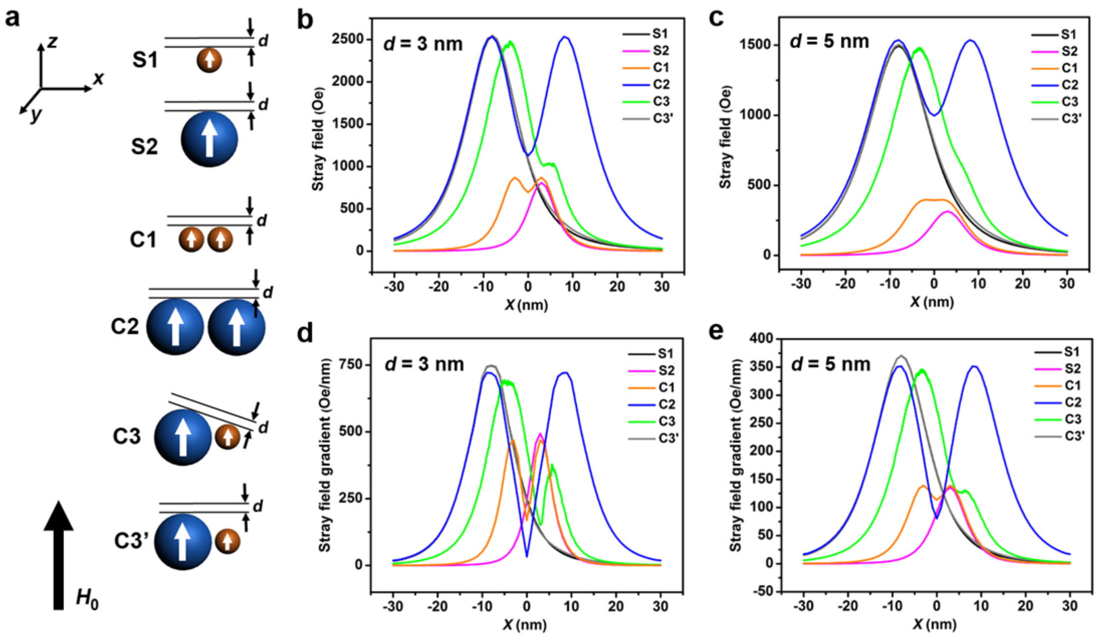

Supplementary Fig. 14 | Additional simulation results for S1, S2, C1, C2, C3, and C3'. (a) Cartoon shows the models for calculating the stray field and field gradient of single IO NPs (IO-5 and IO-15) and IO clusters (C1, C2, C3 and C3'). An external magnetic field (7 T) is applied along the +z direction. (b, c) Calculated results of stray field of these models at out plane distance *d* = 3 and 5 nm, respectively. (d, e) Calculated results of stray field gradient of these models at *d* = 3 and 5 nm, respectively.

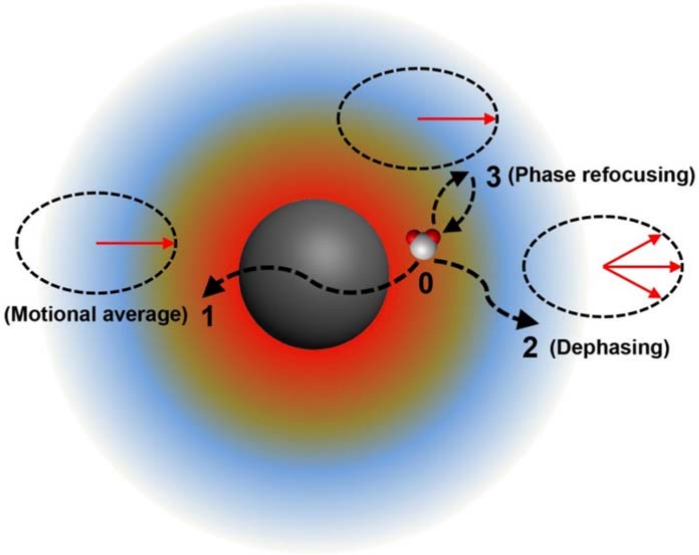

Supplementary Fig. 15 | Schematic model showing water molecule diffusion around a magnetic nanoparticle. Assuming that water molecules at position 0 are at phase focusing state, the diffusion to position 1 would average out the loss of phase coherence and refocus the phase coherence (motional averaging regime). On the other hand, the diffusion to position 2 undergoing an inhomogeneous magnetic field causes the loss of phase coherence (dephasing). The diffusion through an inverse magnetic field is considered to reverse the dephasing event (route 3). This scheme indicates that protons diffusion through the field gradient is the major contributing factor to dephasing and shortening of T_2 relaxation time.

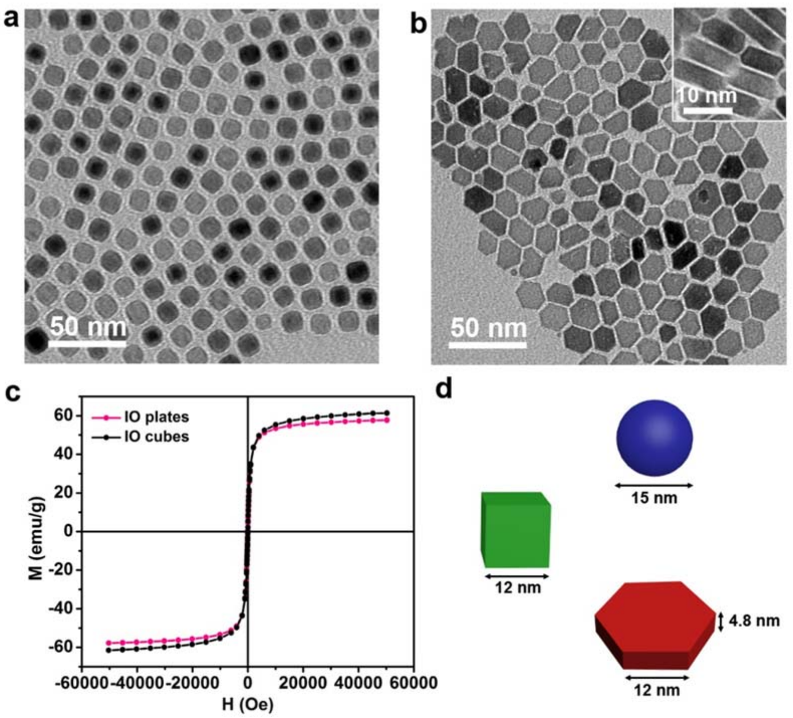

Supplementary Fig. 16 | Characterizations of IO cubes and plates. (a,b) TEM images and (c) magnetic hysteresis curves of IO cubes with side length of 12 nm and IO plates with side length of 12 nm and thickness of 4.8 nm. Inset (b) shows TEM image of vertical nanoplates. (d) Cartoon models showing the physical parameters for IO cubes and plates and IO-15 spheres. These single structures were calculated with equivalent solid volume.

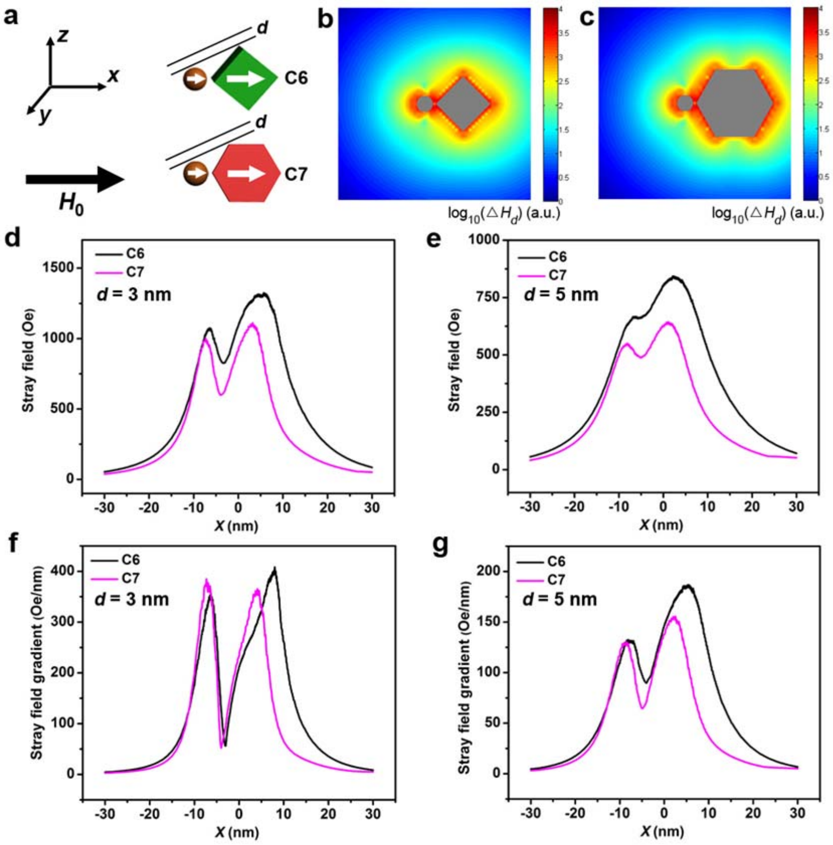

Supplementary Fig. 17 | Simulation results of C6 and C7. (a) Cartoon shows the models C6 and C7 for calculating the stray field and field gradient. An external magnetic field (7 T) is applied along +x direction. (b, c) Simulated stray field gradient for C6 and C7. Color bars represent $\log_{10}(\Delta H_d)$ (a.u.), where H_d is the calculated stray field. (d, e) Calculated stray field of models C6 and C7 at d = 3 and 5 nm, respectively. (f, g) Calculated stray field gradient of models C6 and C7 at d = 3 and 5 nm, respectively.

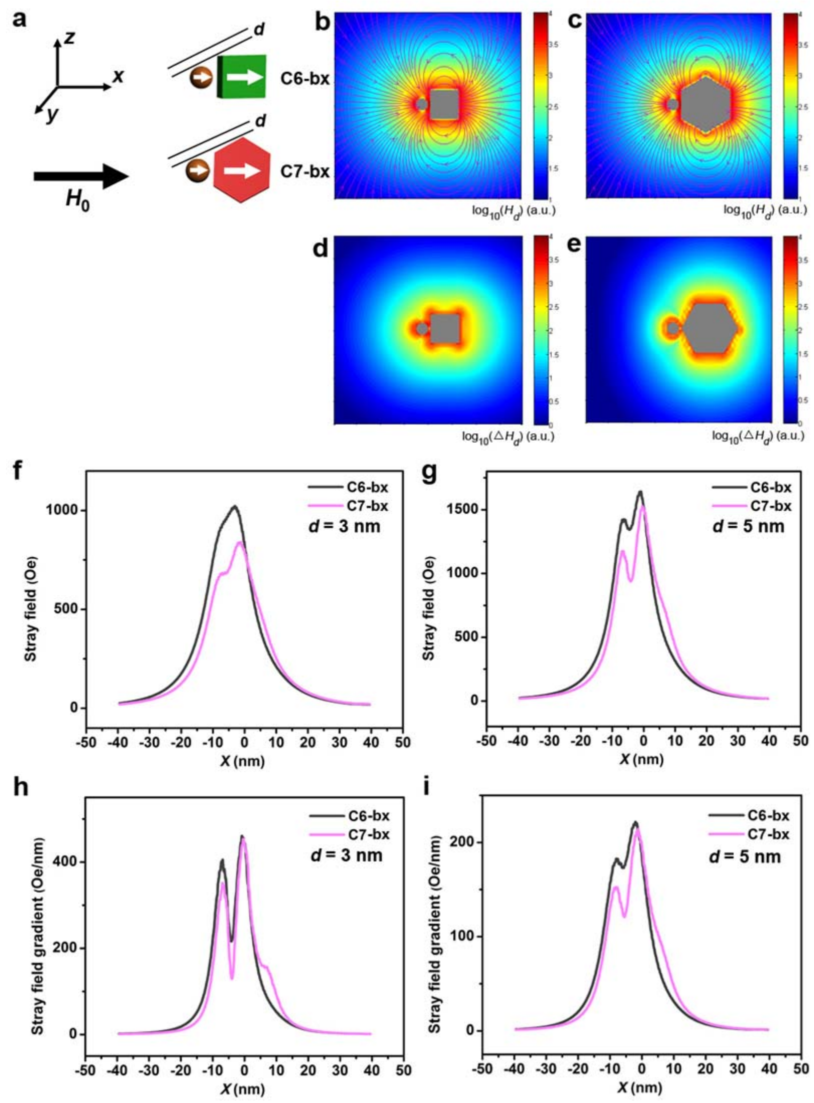

Supplementary Fig. 18 | Additional simulation results of C6 and C7 with different orientations. (a) Cartoon shows the models C6-bx and C7-bx for calculating the stray field and field gradient with an external magnetic field (7 T) applied along +*x* direction. (b-e) Simulated stray field (b,c) and stray field gradient (d,e) for C6-bx and C7-bx, respectively. Color bars represent $\log_{10}(H_d)$ (a.u.) or $\log_{10}(\Delta H_d)$ (a.u.), where H_d is the calculated stray field. (f, g) Calculated stray field of models C6-bx and C7-bx at d = 3 and 5 nm, respectively. (h, i) Calculated stray field gradient of models C6-bx and C7-bx at d = 3 and 5 nm, respectively.

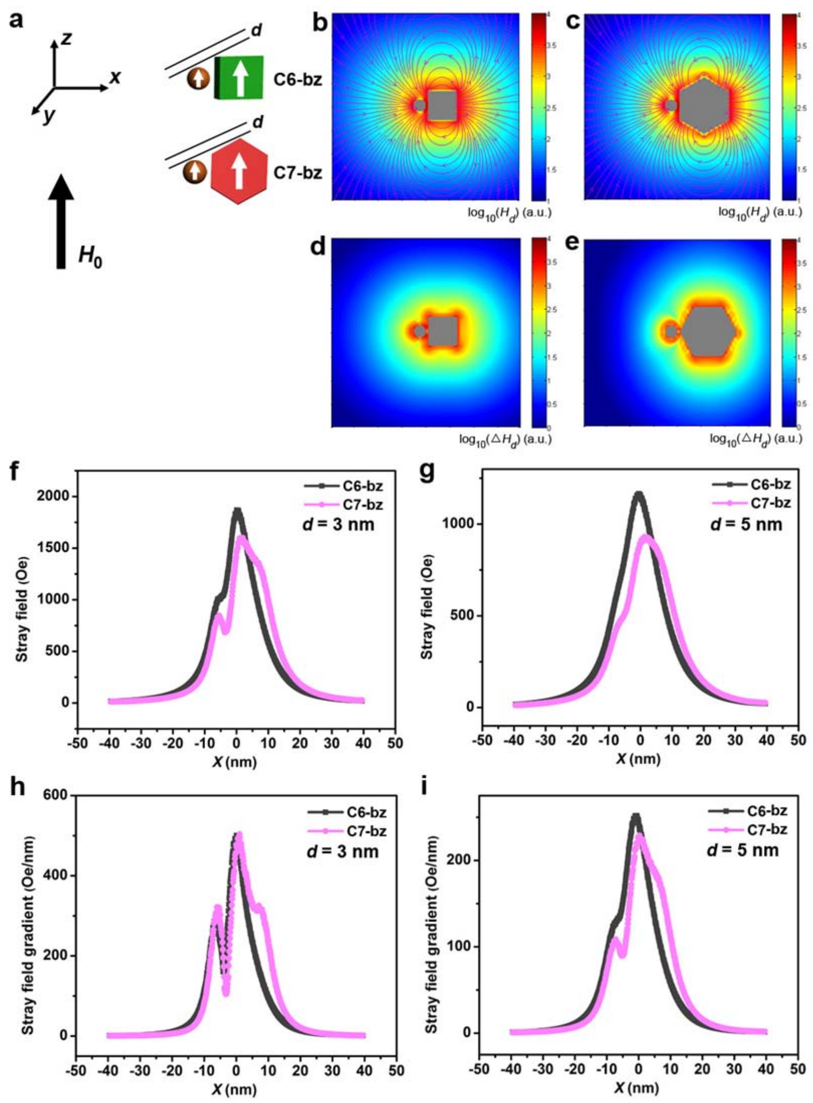

Supplementary Fig. 19 | Additional simulation results of C6 and C7 with different orientations. (a) Cartoon shows the models C6-bz and C7-bz for calculating the stray field and field gradient with an external magnetic field (7 T) applied along +z direction. (b-e) Simulated stray field (b,c) and stray field gradient (d,e) for C6-bz and C7-bz, respectively. Color bars represent $\log_{10}(H_d)$ (a.u.) or $\log_{10}(\Delta H_d)$ (a.u.), where H_d is the calculated stray field. (f, g) Calculated stray field of models C6-bz and C7-bz at d = 3 and 5 nm, respectively. (h, i) Calculated stray field gradient of models C6-bz and C7-bz at d = 3 and 5 nm, respectively.

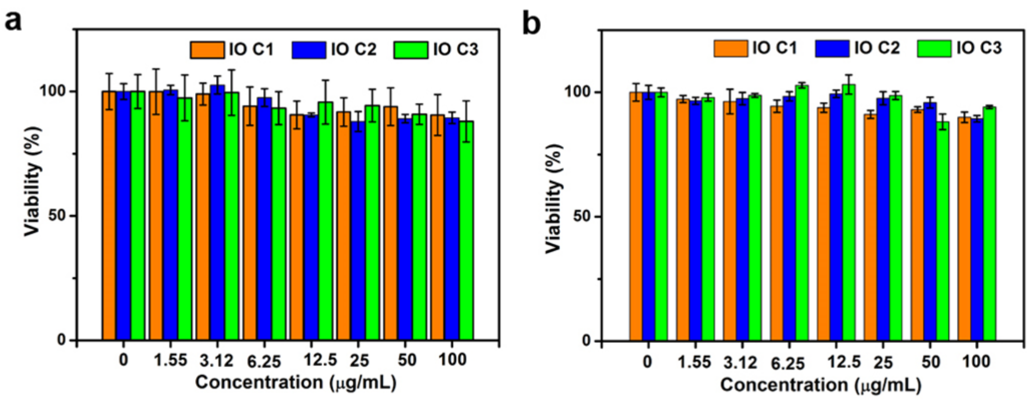

Supplementary Fig. 20 | Cell viability assay of IO clusters C1-C3. Cell viability results of IO clusters C1-C3 on (a) HepG2 and (b) Raw 264.7 cell lines, showing that 90% of both HepG2 and Raw 264.7 cells were viable at concentrations up to 100 µg Fe mL^{-1} after 24 h incubation. The assays were based on cell counting kit-8 (CCK-8) method. Errors are mean values ± s.d.; $n = 3$.

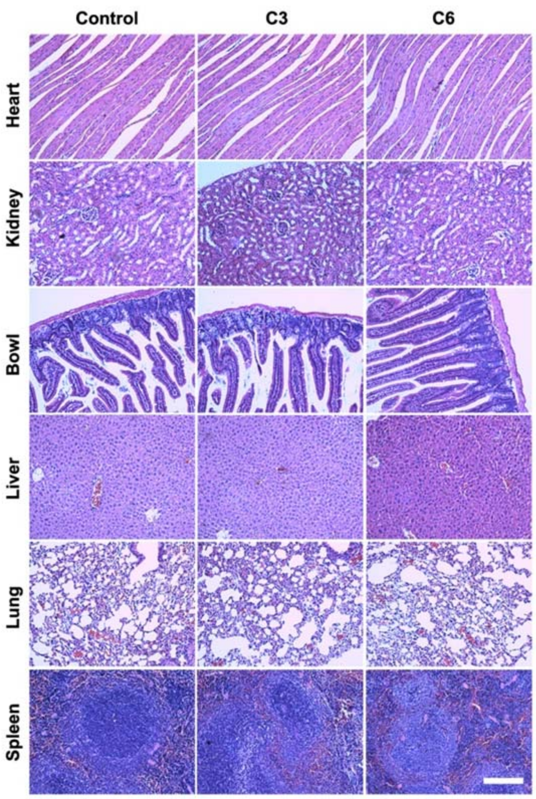

Supplementary Fig. 21 | Representative organ histological analysis of mice. Mice were treated with PBS (control), IO cluster C3 or C6 with a dose of 2.0 mg Fe kg^{-1} mouse body weight through tail vein injection. Organs dissected from mice at 24 h post-injection (p.i.) time. The organs were stained with heamatoxylin and eosin (H&E). No abnormal behavior of mice was found during the treatment time. The major organs of heart, kidney, liver, bowel, lung, and spleen did not exhibit significant microscopic lesions, indcating little acute toxicity of IO clusters C3 and C6 in the living mice model. Scale bar: 100 μm for all images.

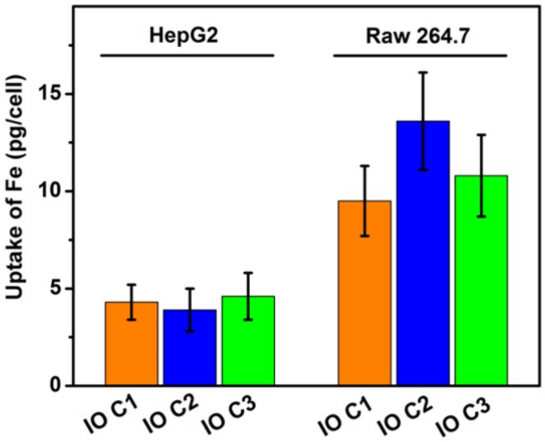

Supplementary Fig. 22 | Cellular uptake analysis of IO clusters at different cell lines. Cellular uptake of IO clusters C1-C3 by HepG2 and macrophage Raw 264.7 cells (~ 10^6) after 4 h incubation at a concentration of 50 μg mL^{-1} with respect to iron mass. Each point was obtained as the mean of triplicate measurements ± s.d. The concentrations were determined by inductively coupled plasma atomic emission spectroscopy (ICP-AES). Errors are mean values ± s.d.; n = 3.

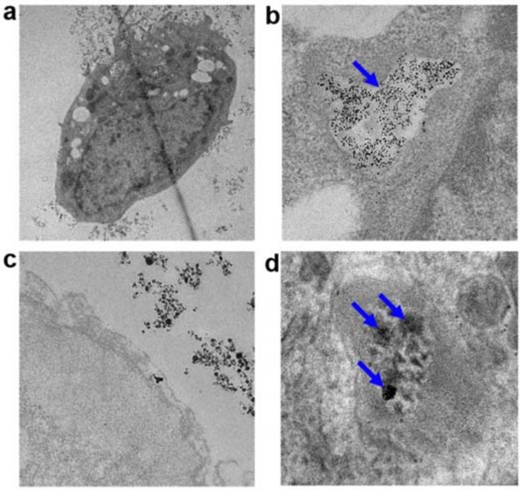

Supplementary Fig. 23 | Sectional cell TEM images. TEM images of RAW 264.7 cells after being incubated with (a,b) single IO-15 NPs or (c,d) IO cluster C2. Blue arrows indicate IO NPs in endocytotic vesicles.

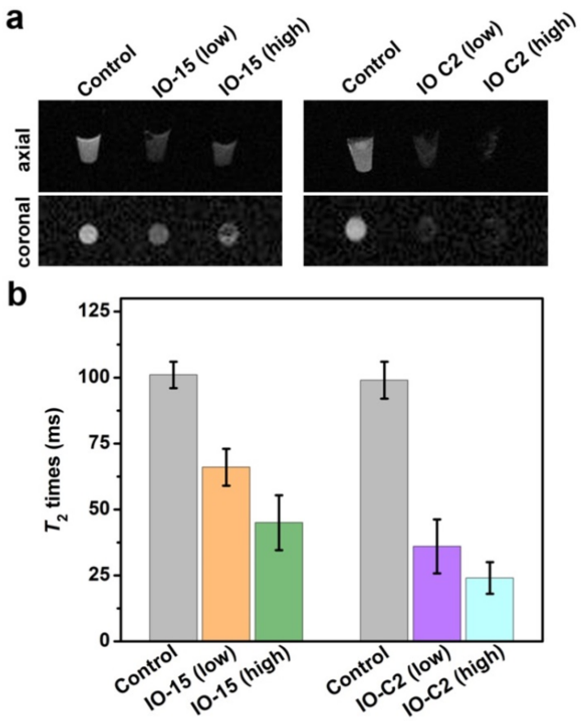

Supplementary Fig. 24 | *In vitro* cell MRI study conducted on a 7 T MRI scanner. (a) MR phantom and (b) T_2 relaxation times of Raw 264.7 cells after being incubated with different concentrations of single IO-15 (left) and IO C2 (right) samples for 4 h. The control, low, and high indicate the final concentrations of 0, 2, and 5 μg mL^{-1} with respect to iron mass, respectively. Each well contains ~ $5*10^6$ cells mixed with 1% of agarose gel. Errors are mean values ± s.d.; $n = 3$.

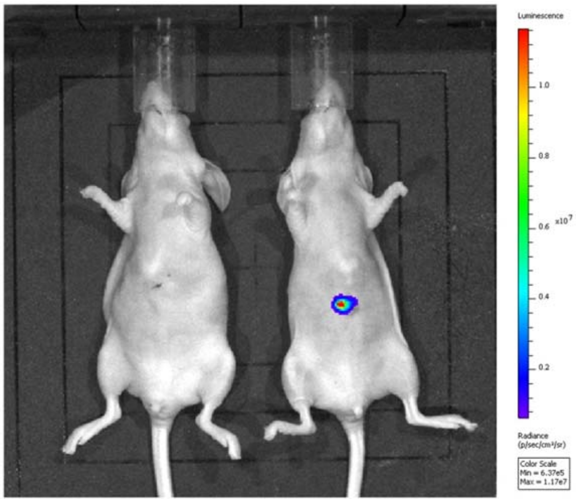

Supplementary Fig. 25 | Bioluminescence imaging of tumor bearing mice. The orthotopic liver tumor models were established by HepG2 cells with genetically encoded firefly luciferase. The image was acquired after intravenous injection of a substrate luciferin to allow bioluminescence recovery by luciferase specifically at the tumor region (right). No bioluminescence was observed in a control mouse without HepG2 tumor (left).

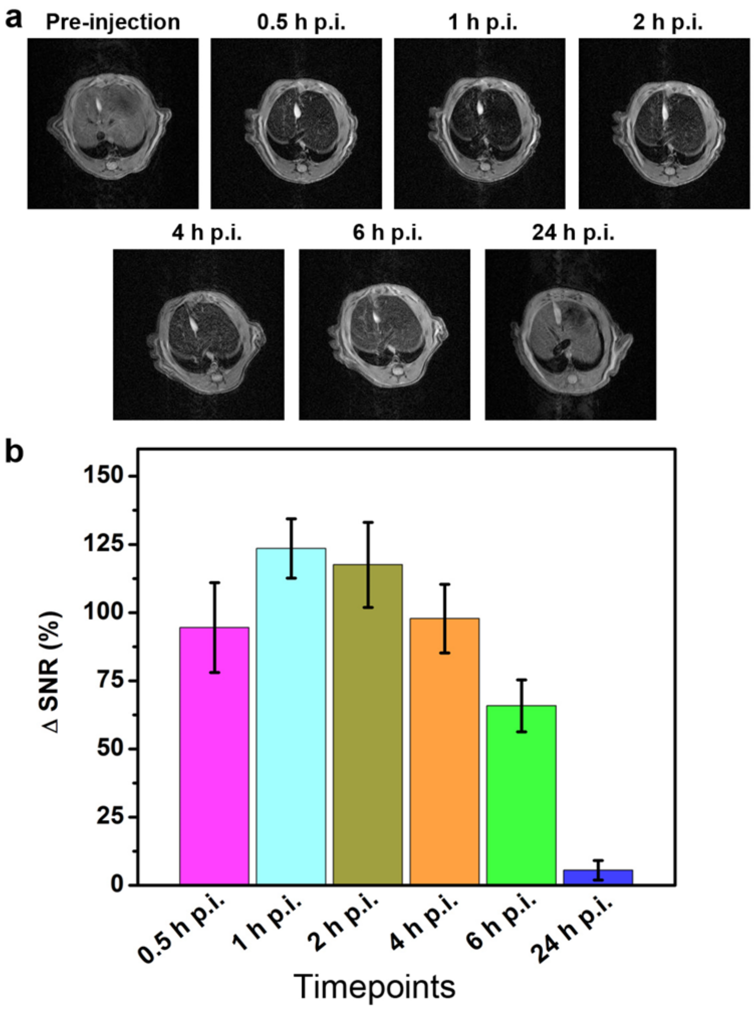

Supplementary Fig. 26 | *In vivo* MRI of mouse liver. (a) MR images of mouse liver before and after intravenous injection of IO C3 with a dose of 1.0 mg kg^{-1} with respect to iron mass to mouse body weight. The axial MR images of liver at time-points of 0.5, 1, 2, 4, 6, and 24 h post-injection (p.i.) were acquired to compare with the image of pre-injection. (b) Quantitative analysis of the changes of signal-to-noise (ΔSNR) at different p.i. time-points, showing that the maximum ΔSNR was attained at around 1 h p.i. in liver. Errors are mean values ± s.d.; $n = 3$.

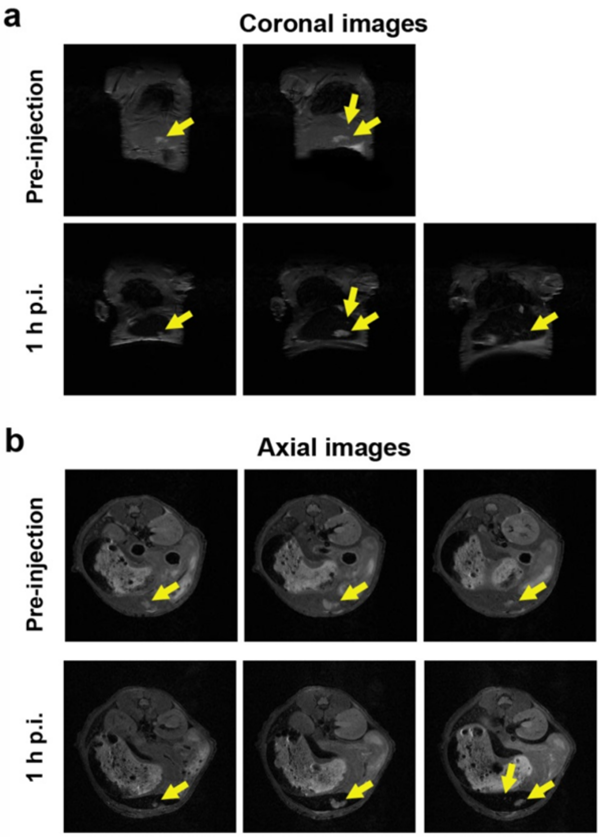

Supplementary Fig. 27 | Additional *in vivo* T_2 MR images of liver tumor model. MR images of mouse liver bearing an orthotopic liver tumor (HepG2) using IO cluster C3 as contrast agents on a 7 T MRI scanner. The IO cluster C3 was injected through intravenous route with a dose to iron concentration of 1.0 mg Fe kg^{-1} mouse body weight. All of the MR images at (a) coronal and (b) axial planes with slices containing tumor (or small lesion) are presented. Yellow arrows indicate liver tumor (or small lesion).

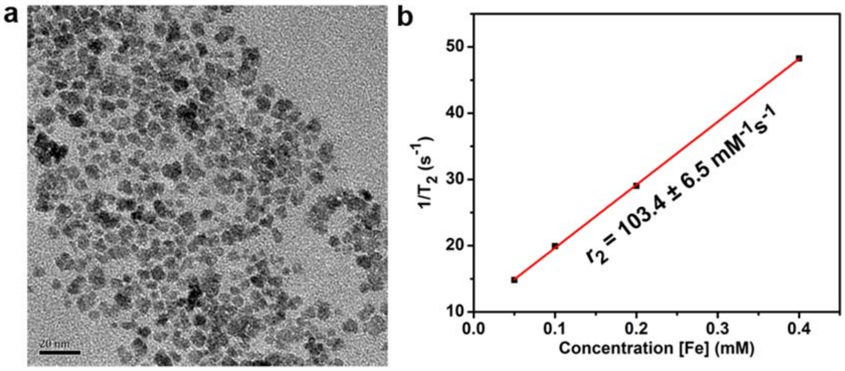

Supplementary Fig. 28 | Characterizations of ferumoxytol (Feraheme). (a) TEM images and (b) T_2 relaxivity measured on a 7 T MRI scanner, showing the r_2 value of 103.4 ± 6.5 $mM^{-1}s^{-1}$ with respect to concentration of iron ions. Errors are mean values ± s.d.; $n = 3$.

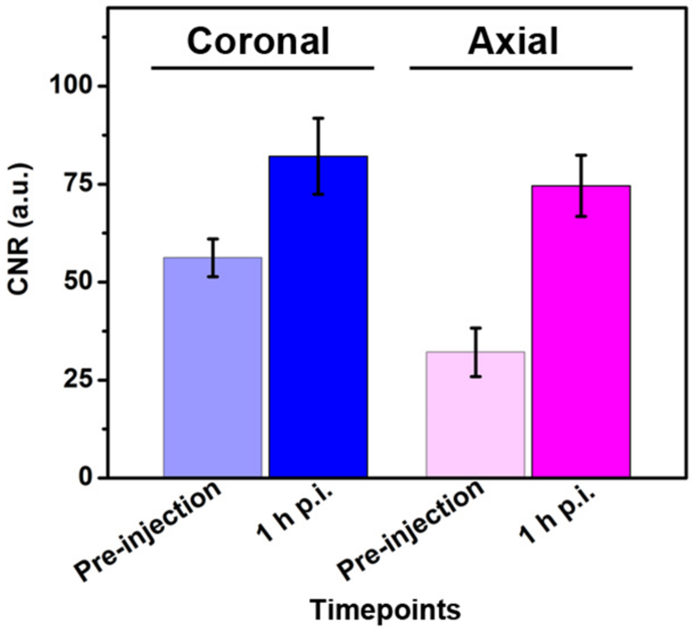

Supplementary Fig. 29 | Quantitative analysis of contrast-to-noise ratios of tumor. The analysis was based on pre-injection and 1 h post-injection (p.i.) images of mouse liver bearing an orthotopic liver tumor (HepG2) as described in the Figure 5. Ferumoxytol was injected through intravenous route at a dose of 1.0 mg Fe kg^{-1} to mouse body weight and contrast-enhanced MRI study was conducted on a 7 T scanner. Errors are mean values ± s.d.; n = 3.

Supplementary Methods

Synthesis of iron oxide nanoparticles. Iron oxide nanoparticles (IO NPs) with diameter of 5 nm were obtained following procedures reported elsewhere (Supplementary Refs. 1 and 2). IO NPs with diameter of 15 nm were modified from a previously developed method. To obtain IO-5 NPs, 353 mg of $Fe(acac)_3$, 1 mL of oleic acid, 1 mL of oleyl amine, 1.25 g of 1,2-hexadecanediol, and 10 mL of benzyl ether were mixed in a flask, allowing for degassing with N_2 under 100 °C for 20 min. The system was then heated under reflux and maintained for 30 min before cooling to room temperature. The product was collected by centrifugation with the addition of ethyl alcohol and purified with a mixture of hexane and ethyl alcohol. The final product of IO-5 NPs was dispersed in hexane for further use. To obtain IO-15 NPs, 900 mg of iron oleate, 0.16 mL of oleic acid, and 15 mL of 1-octadecene were mixed in a flask and degassed with N_2. The system was heated under reflux and maintained for 1 h. After cooling to room temperature, excess of isopropanol was added to precipitate the product. The final product was obtained by centrifugation and dispersed in hexane for further use.

For the synthesis of IO nanoplates, 0.9 g (1 mmol) iron oleate complex was dissolved in 10 mL benzyl ether, with the addition of 0.16 mL (0.5 mmol) oleic acid and 15 mg (0.1 mmol) sodium oleate. The solution was kept at 120 °C for 20 min before reaching reflux temperature, and was allowed to react for 1 h before cooling to room temperature. The products were obtained by addition of 50 mL ethanol and were collected by centrifugation at 7000 rpm for 10 min. After washing with ethanol twice, the precipitation was dispersed in hexane. The synthesis of IO cubes was performed as follows: 900 mg (1 mmol) of iron oleate was dissolved in 15 mL of TOA in the presence of 159 μL (0.5 mmol) of oleic acid. After replacement with a nitrogen atmosphere, the flask was then heated to

320 °C with a heating rate of about 10-15 °C per min and maintained at 320 °C for 2 h. The heating source was then removed to allow the system to cool to room temperature. The products were obtained by a similar procedure as above and dispersed in hexane. The samples for magnetization measurements were subjected to solvent washing (3 times) and metal bath drying (100 °C) to obtain dry powder samples.

Synthesis of Pphosphonated poly(ethylene glycol) and ATRP initiator. The ATRP initiator BiBEP was synthesized according to the method reported by Matyjaszewski *et al.* (Supplementary Ref. 3). To a solution of dimethyl(2-hydroxyethyl) phosphonate (1 g, 6.5 mmol) in methylene chloride (10 mL), 0.9 mL (7 mmol) of bromotrimethylsilane was sequentially added at 0 ºC. After 1 h, the temperature was raised to ambient temperature for a further 12 h. The solvent and volatile residues were removed then by evaporation, and methanolysis of the silylated intermediate was realized by adding an excess of methanol (5 mL) and stirred at room temperature for another 12 h. The solvent was evaporated to provide a quantitative yield of a slightly yellowish oil (2-hydroxyethyl) phosphonate after washing with ether. To prepare phosphonated poly(ethylene glycol) (PEG-P), PEG-COOH (0.5 g, 0.1 mmol) and (2-hydroxyethyl) phosphonate (0.038 g, 0.3 mmol) were dissolved in DMSO (5 mL), and DCC (0.041 g, 0.2 mmol) was slowly added to it. After the solution was stirred for 30 min at room temperature in the dark, DMAP (0.003 g, 0.025 mmol) in 0.5 mL DMSO was added. The mixture was stirred overnight in the dark at room temperature, filtered to remove the insoluble byproduct, dicyclohexylurea, and was precipitated using diethyl ether. The PEG-P was collected by filtration, washed with a mixture of dry diethyl ether and acetone (4:1) and dried under vacuum.

Calculation of the graft density of PMMA on the IO nanoparticles surface. Given the density (ρ)

of the IO NPs (Fe_3O_4) is 5.18 g/cm^3, the molar mass of the IO NP ($M_{IO\ NP}$) can be calculated using Equation 1, where r is the radius of the IO NP. Combining the molar mass of the IO NP, the molecular weight of polymer brush ($M_{polymer}$) and the weight fraction obtained in TGA analysis, the average number of polymer grafts can be calculated by Equation 2, where $W_{polymer}$ is the weight fraction of the organic part and $W_{IO\ NP}$ is the weight fraction of IO NP. For the IO@PMMA nanoparticle, the molecular weight of M_{PMMA} is 25.5 kDa and the organic weight fraction is 60%. The result gives a number of 212 PMMA chains per NP with graft density of 0.40 chain/nm^2.

$$M_{\mathrm{IO\ NP}} = (\rho V_{\mathrm{IO\ NP}}) = \left(\rho \frac{4}{3}\pi r^3\right) \qquad (1)$$

$$N_{\mathrm{grafts\ per\ IO\ NP}} = \left(\frac{W_{\mathrm{polymer}} / M_{\mathrm{Polymer}}}{W_{\mathrm{IO\ NP}} / M_{\mathrm{IO\ NP}}} \right) \qquad (2)$$

Preparation of DMSA coated IO NPs. We prepared DMSA coated IO NPs through a ligand exchange process (Supplementary Ref. 4). Briefly, excess DMSA (20 mg) was dissolved in 10 mL ultra-pure water in a three-neck flask. The as-prepared IO NPs (100 µmol) dispersed in hexane were added to the flask. The solution was then heated to reflux for 2 h. After cooling to room temperature, acetone was added to the lower layer and purified by centrifugation. The final products were redispered in water and stored at 4 °C for further use.

Supplementary Table 1 Summary of r_2 values of single IO-5 and IO-15 NPs, and IO clusters C1-C5 measured on a 7 T MRI scanner. The effective radius for IO cubes and IO plates are about 20 and 28 nm when modeling them as spheres. Mean values ± s.d.; n = 3.

Samples	Saturated magnetization (emu g^{-1})	Diameter (nm)	r_2 ($mM^{-1}s^{-1}$)	r_1 ($mM^{-1}s^{-1}$)	r_2/r_1
**IO-5**	43.18	5.2 ± 0.2	70.2 ± 5.7	1.38 ± 0.3	50.9
**IO-15**	65.10	15.1 ± 0.3	127.4 ± 3.4	1.17 ± 0.2	108.9
**IO C1**	40.28	115.5 ± 10.4	231.6 ± 9.3	2.11 ± 0.2	109.7
**IO C2**	60.37	127.8 ± 13.4	358.3 ± 14.2	0.8 ± 0.2	447.9
**IO C3**	53.69	129.2 ± 11.2	533.4 ± 13.2	0.92 ± 0.1	579.8
**IO C4**	54.73	145.8 ± 23.5	515.3 ± 18.9	1.56 ± 0.2	330.3
**IO C5**	50.22	238.9 ± 36.7	445.8 ± 24.6	1.35 ± 0.3	330.2
**IO cubes**	61.31	n/a	157.9 ± 6.9	0.9 ± 0.1	175.4
**IO plates**	57.60	n/a	204.5 ± 11.4	1.44 ± 0.2	142.0
**IO C6**	51.23	85.7 ± 13.9	589.3 ± 26.8	2.24 ± 0.2	263.1
**IO C7**	51.38	91.6 ± 15.3	487.7 ± 21.5	2.76 ± 0.5	176.7

Supplementary References

1. Park, J. et al. Ultra-large-scale syntheses of monodisperse nanocrystals. *Nat. Mater.* **3**, 891-895 (2004).

2. Zhou, Z. et al. Anisotropic shaped iron oxide nanostructures: controlled synthesis and proton relaxation shortening effects. *Chem. Mater.* **27**, 3505-3515 (2015).

3. Dong, H. et al. Recyclable antibacterial magnetic nanoparticles grafted with quaternized poly(2-(dimethylamino)ethyl methacrylate) brushes. *Biomacromolecules* **12**, 1305-1311 (2011).

4. Zhou, Z. et al. Engineered iron-oxide-based nanoparticles as enhanced T1 contrast agents for efficient tumor imaging. *ACS Nano* **7**, 3287-3296 (2013).
